# Discovery of Novel Metalloenzyme Inhibitors Based on Property Characterization: Strategy and Application for HDAC1 Inhibitors

**DOI:** 10.3390/molecules29051096

**Published:** 2024-02-29

**Authors:** Lu Zhang, Yajun Yang, Ying Yang, Zhiyan Xiao

**Affiliations:** 1 Beijing Key Laboratory of Active Substance Discovery and Druggability Evaluation, Institute of Materia Medica, Chinese Academy of Medical Sciences and Peking Union Medical College, Beijing 100050, China; zhanglu6@tj.gov.cn (L.Z.); yangyajun@imm.ac.cn (Y.Y.); yangying@imm.ac.cn (Y.Y.); 2 Department of Toxicology, Tianjin Centers for Disease Control and Prevention, Tianjin 300011, China; 3 State Key Laboratory of Digestive Health, Institute of Materia Medica, Chinese Academy of Medical Sciences and Peking Union Medical College, Beijing 100050, China

**Keywords:** metalloenzyme inhibitors, metal-binding fragments, property characterization, histone deacetylase, virtual screening

## Abstract

Metalloenzymes are ubiquitously present in the human body and are relevant to a variety of diseases. However, the development of metalloenzyme inhibitors is limited by low specificity and poor drug-likeness associated with metal-binding fragments (MBFs). A generalized drug discovery strategy was established, which is characterized by the property characterization of zinc-dependent metalloenzyme inhibitors (ZnMIs). Fifteen potential Zn^2+^-binding fragments (ZnBFs) were identified, and a customized pharmacophore feature was defined based on these ZnBFs. The customized feature was set as a required feature and applied to a search for novel inhibitors for histone deacetylase 1 (HDAC1). Ten potential HDAC1 inhibitors were recognized, and one of them (compound **9**) was a known potent HDAC1 inhibitor. The results demonstrated the effectiveness of our strategy to identify novel inhibitors for zinc-dependent metalloenzymes.

## 1. Introduction

Metalloenzymes are involved in various biological processes, including epigenetic regulation, immune regulation, antimicrobial resistance and metabolism. Therefore, metalloenzymes are associated with a variety of diseases such as cancer, arthritis, cardiovascular disease, glaucoma, Alzheimer’s and AIDS [1,2]. At least 558 out of the 1371 Enzyme Commission (EC) entries recorded in MACiE databases are attributed to metal-dependent enzymes, which represents a significant portion of the enzyme family [3]. The extensive association with human diseases and the large number of known metalloenzymes have stimulated intense research interest in the discovery of novel metalloenzyme inhibitors as potential therapeutic agents.

**Figure 1:**
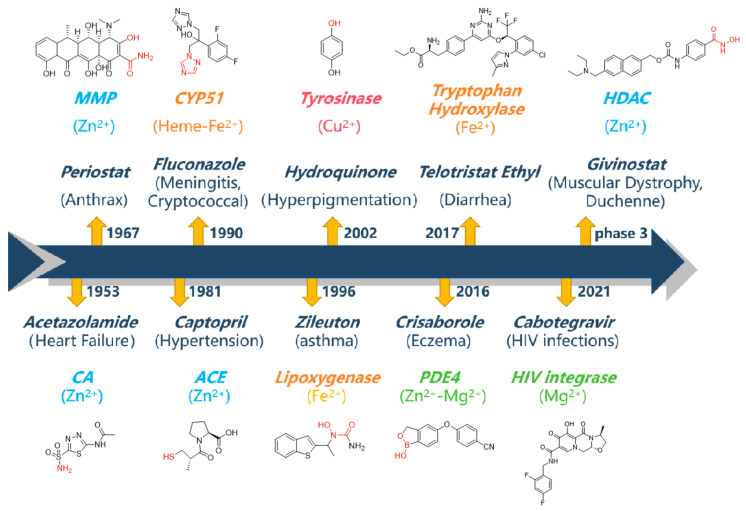
Representative metalloenzyme inhibitors as therapeutical drugs. Metal-binding fragments are represented in red. (Data from Chembl database, https://www.ebi.ac.uk/chembl/, accessed on 15 February 2024).

To date, the U.S. Food and Drug Administration (FDA) has approved more than 60 small-molecule metalloenzyme inhibitors, and representative drugs are shown in Figure 1 [4,5]. Currently, more than 1500 small-molecule drugs are on the market, the proportion of metalloenzyme inhibitors is only about 4%, which is surprisingly insignificant compared to the proportion of metalloenzymes in all enzymes (40%). This disparity implies that the development of therapeutics targeting metalloenzymes can be demanding and challenging [5]. As demonstrated by the representative metalloenzyme inhibitors (Figure 1), the vast majority of these inhibitors present a metal-binding fragment (MBF), which plays a key anchoring role in enzyme–inhibitor interaction [6]. The identification of MBFs to achieve structural diversity and favorable physicochemical properties has become a bottleneck in the development of metalloenzyme inhibitors. Take the Zn^2+^-dependent metalloenzyme as an example. Hydroxamic acid is a preferred chelator for Zn^2+^. As a well-known MBF, hydroxamic acid is readily introduced to the inhibitor structures and can generally ensure potent binding. Therefore, it presents ubiquitously in inhibitors targeting various metalloenzymes, including matrix metalloproteins (MMPs), histone deacetylases (HDACs), and many others. However, it also associates with disadvantages such as poor selectivity and poor pharmacokinetic profiles. Hence, it might be responsible for the severe side effects and insufficient efficacy related to metalloenzyme inhibitors [7,8].

Currently, the discovery of novel metalloenzyme inhibitors typically involves screening chemical libraries of structurally diverse chelators against targets of interest for potential hits. Dick et al. [9] synthesized 24 metal-binding isosteres based on the structure of picolinic acid, and approximately half of the isosteres exhibited inhibitory activity similar to picolinic acid. The results demonstrate that the isosteric replacement of MBFs is a feasible strategy to generate novel metalloenzyme inhibitors. Agrawal et al. [10] constructed a chelator fragment library based on a variety of metal-binding groups and the library was screened against MMP-2. A hit was identified, and an expanded library derived from the hit was then synthesized, which afford high-affinity hits against MMP-2. These approaches did facilitate the discovery of novel metalloenzyme inhibitors. However, the chelator libraries were generated based on existing metal binding fragments and were unlikely to provide novel MBFs. Therefore, innovative strategies and approaches are urgently needed to expand the diversity of MBFs.

Recent efforts to identify novel MBFs have resorted to computational approaches, for which the appropriate parameterization of metal ions is the major challenge. Most currently available docking programs cannot accurately model the interaction between inhibitors and metal ions. Researchers have striven to improve the specificity and accuracy of docking, and strategies including customizing field parameters [11], predefining coordination configurations [12], establishing specific scoring functions [13], and re-scoring based on docking consistency [14] have been actively pursued. Furthermore, metal–ligand interactions are often described as hydrogen-bonding interactions in pharmacophore characterization, which results in poor model specificity [15,16]. Currently available methods to improve model specificity mainly include establishing specific fragment queries [16] and constructing specific metal-binding pharmacophores [17]. In addition, QSAR methods have also been applied to metalloenzyme inhibitor characterization. It is worth noting that the introduction of appropriate quantum chemical descriptors calculated by density functional theory (DFT), such as ionization potential, electron attraction or affinity, charges on the atoms of the molecule, multipole moments, and polarization, can effectively improve the performance of QSAR models in predicting compound activity [18,19].

In our previous studies [20,21], novel indoleamine 2, 3-dioxygenase (IDO) 1 and xanthine oxidase (XO) inhibitors were successfully identified through fragment recognition and property characterization of the MBFs. To search for novel XO inhibitors [21], we applied the enhanced characterization of the molybdopterin-binding group (MBG) with DFT-based physical, chemical, and topological properties. Two novel potent XO inhibitors with novel MBGs were thus recognized and showed IC_50_ values of 23 nM and 26 nM, respectively. Such results encouraged us to generalize the property characterization strategy to discover novel MBFs and metalloenzyme inhibitors for certain subfamilies of metalloenzymes. Accordingly, we extended our property characterization strategy to zinc-dependent metalloenzymes herein. Briefly, zinc-dependent metalloenzyme inhibitors (ZnMIs) were characterized and key electronical descriptors related to Zn^2+^-binding fragments (ZnBFs) were revealed. The fragment libraries were then screened based on the classification model to identify potential ZnBFs. Finally, the reliability of the identified ZnBFs was verified using histone deacetylase (HDAC) 1 as a validation target. A substructure search for potential ZnMIs successfully identified a known and potent HDAC1 inhibitor, which supports the reliability and validity of the screening strategy.

## 2. Results and Discussion

### 2.1. Dataset Collection and Descriptor Selection

PDB complex entries for 757 Zn^2+^-dependent metalloenzymes were collected with their binding ligands and K_i_ information (represented in nM). For ligands with inconsistent K_i_ values in different PDB entries, the activity values were represented by the average values of the lg (K_i_) when the lg (K_i_) difference was less than or equal to 1, or discarded when the lg (K_i_) difference was larger than 1. A total of 523 ligands with lg (K_i_) values ranging from −4 to 7 were retained. These ligands were then divided into a high-activity group (lg (K_i_) < 2) and low-activity group (lg (K_i_) > 3). In each group, one of the two ligands with pair similarity by fingerprint MACCs greater than 0.75 was arbitrarily removed to reduce redundancy. A dataset with 131 high-activity and 77 low-activity ligands was gathered. A total of 165 ligands in the dataset were randomly selected as the modeling set, and the remaining 43 ligands were used as the external test set.

**Figure 2:**
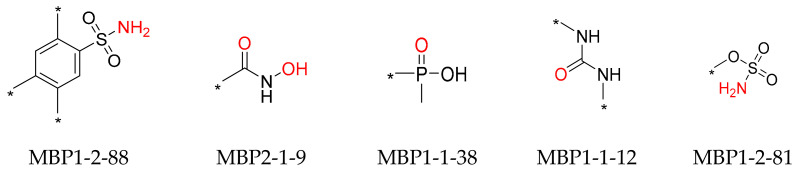
Structures of the five most common zinc-binding fragments presented in 135 high-activity ligands. Metal-binding atoms are shown in red, and variable fragments are incorporated at the sites represented by *.

The zinc-binding fragments in the 131 high-activity ligands were analyzed and are shown in Table S1. Five zinc-binding fragments, namely MBP1-2-88, MBP2-1-9, MBP1-1-38, MBP1-1-12, and MBP1-2-81 (the MBP numbers were quoted from reference [22]), were identified as the most frequently presented fragments (more than five times) and are shown in Figure 2. Four fragments bind to Zn^2+^ in a monodentate coordination manner through the oxygen atom of the phosphoric acid or urea group and the nitrogen atom of the sulfonamide group. Yet, the hydroxamic acid fragment binds to Zn^2+^ in a bidentate coordination manner through the two oxygen atoms.

A total of 3109 descriptors were calculated based on the entire ligand structures in their original form presented in the complex structures. Descriptors with zero variance or with a correlation coefficient to lg (K_i_) less than 0.15 were first removed. For descriptors with pairwise correlation coefficient values greater than 0.8, one of the two descriptors with a lower correlation coefficient to lg (K_i_) was deleted. After initial screening, 125 descriptors were retained. Four different descriptor selection methods, including Cfs subset evaluation (Cfs), gain ratio attribute evaluation (GR), wrapper subset evaluation (Wrapper), and Cfs subset evaluation combined with wrapper subset evaluation (Cfs&wrapper), were applied to select four descriptor sets with 69, 31, 71, and 34 descriptors, respectively.

### 2.2. Construction of Classification Models

Based on the four selected descriptor sets, the modeling set was further randomly divided into a training set and an internal test set by cluster analysis with a ratio of 7:3. Eight algorithms including Naive Bayes (NB), IBK, J48 [23], Attribute Selection Classifier (ASC)_NB, ASC_IBK, ASC_J48, ZeroR, and OneR were used in model construction. The accuracy of the constructed model was evaluated by ten-fold cross-validation. The validation was repeated ten times using different random seeds, and the average classification accuracy was returned to evaluate the performance of different classification methods.

The modeling results of the IBK algorithm were mainly affected by the K value, which represented the number of adjacent samples used to predict the category of unknown samples. IBK models with different K values were constructed, and the results are shown in Table S2 and Figure S1A. At the beginning, as the K value increased, the accuracy gradually improved due to the reduction in noise interference. However, when the K value increased to a certain extent, the increase in K value resulted in inaccurate classification and even worse accuracy. Generally, the modeling accuracy of the IBK algorithm was slightly higher than that of the ASC_IBK algorithm. Since the IBK algorithm is based on the similarity between samples, it is reasonable that IBK is not sensitive to the selection of attributes. The best prediction accuracy was 76.50%, which was produced by modeling with the Cfs&Wrapper descriptor selection method and a K value of 25 (Table 1).

When modeling with the J48 algorithm, different pruning methods with variable minimum numbers of instances (Minimum Obj) were adopted to reduce noise interference. The modeling results are shown in Figure S1B and Table S3. As the Minimum Obj value increased, the accuracy first increased and then decreased. In addition, the accuracy of the models constructed by the ASC_J48 algorithm was generally better than that of the J48 algorithm. The results show that the J48 algorithm is sensitive to attribute selection, which is consistent with the fact that the J48 algorithm is based on attribute values. The best predictive accuracy was 75.43%, which was obtained by modeling with the GR descriptor selection method and Minimum Obj of 4 (Table 1).

The remaining modeling algorithms (ASC_NB, NB, ZeroR, and OneR algorithms) were also used, and the best accuracy rates were 78.52%, 82.82%, 63.82%, and 72.01%, respectively. Among them, 63.82% was the baseline accuracy as no attributes were referenced, and the classification was totally random. This indicates that the two groups of compounds are essentially balanced in our modeling set (Table 1).

Among the eight modeling algorithms, the NB and ASC_NB algorithms showed the best performance, and predictive accuracy higher than 75% was achieved, which was over 10% higher than the baseline accuracy (63.82%). The NB algorithm can effectively predict unknown instances based on the frequency of sample distribution and has strong anti-interference ability. The modeling accuracy rates of the IBK and ASC_IBK algorithms were higher than those of the J48 and ASC_J48 algorithms, indicating that IBK has better anti-interference ability. The predictive accuracy of the OneR algorithm, modeled with only one attribute, was still better than that of the ZeroR algorithm. Such results suggested that the selection of attributes was reasonable.

### 2.3. Classification Model Validation

Ten different groups of training and internal test sets were randomly generated and modeled using the NB and ASC_NB algorithm, respectively. The accuracy of the model was evaluated through a 10-fold cross-validation method and further validated through internal and external test sets. The validation results are shown in Table 2. When modeling with the Cfs&Wrapper descriptor selection method and NB algorithm (referred to as Cfs&Wrapper-NB), the predictive accuracy of the training set and the internal test set exceeded 80%, and the predictive accuracy of the external test set reached 72.5%.

Then, the ten models established by the Cfs&Wrapper-NB method were further analyzed. Model 5 showed the best predictive accuracy on the external test set, and the AUC values of the ROC curve derived from the external test set reached 0.80 (Table 3). Therefore, it was used for the subsequent screening of fragment libraries. The structures and relevant information regarding the training, internal test, and external test sets used for the construction and validation of Model 5 are shown in Figure S2 and Table S4.

The false-positive molecules predicted by Model 5 were then analyzed, and their structures are shown in Figure S3. Most of the false-positive molecules were sulfonamides and hydroxamic acid analogs, which may attribute to the higher proportion of these two structural classes in the high-activity group.

The learning curve derived from Model 5 is shown in Figure S4. When the sample size exceeded 80%, the model accuracy slowly increased and reached a stable level. This result indicates that the sample size is sufficient and the model is reliable.

### 2.4. Interpretation of the Model

The 34 descriptors selected by the Cfs&Wrapper method used in Model 5 were considered to be important descriptors for ZnMIs. The correlation coefficients and statistical significance between the 34 descriptors were then analyzed, and their eigenvalues and classification are shown in Table S5. Sixteen of the thirty-four descriptors had absolute correlation coefficients greater than 0.3 and were statistically significant in different categories by the Mann–Whitney U test. These descriptors were considered to be highly correlated with the activity of ZnMIs. In particular, the descriptors related to electronic properties are considered to be highly relevant to MBFs due to the critical role of electronic natures in MBF-Zn^2+^ interaction. Eight descriptors related to electronic properties were then further analyzed, as shown in Table S6. The correlation coefficients of these descriptors were 0.44, 0.40, 0.36, 0.35, 0.35, 0.34, 0.31, and 0.30, respectively.

The data distribution of the eight descriptors for the two classes of compounds is shown in Figure S5. JGI9 and JGI8 are topological charge descriptors. BCUTp-1h represents the highest polarizability weighted by BCUTS. PEOE_VSA-4, PEOE_VSA+1, and PEOE_VSA-0 are local charge descriptors representing the sum of vi, where the range of qi is [−0.25, −0.20), [0.05, 0.10), and [−0.05, 0.00), respectively. The result suggests that higher polarizability, more local negative charges, or fewer positive charges would lead to stronger bonding with metals. In addition, nAtomP and HBA_Count are related to the number of atoms in the maximum π system and the number of hydrogen-bond-accepting groups in the molecule, respectively. The identification of these descriptors revealed key structural features contributing to metal binding, and the presence of larger π systems and more hydrogen bond acceptors might be favorable for metal binding. These results are consistent with the previous observation that the higher the electron-donating capacity of the ligand, the stronger the binding ability to metalloenzymes [24]. All in all, these eight descriptors significantly affect the classification results and could enhance the characterization of MBFs.

### 2.5. Fragment Library Screening

A total of 73,212 small-size molecules were collected from commercial chemical libraries (such as Specs, Topscience, and J&K) and our in-house library of synthetic intermediates as a potential fragment library. The molecules were first predicted by Model 5, and those predicted to be highly active were further analyzed. Only molecules with electronic descriptor values in the range of (μ − 1.96σ ~ μ + 1.96σ) were retained, where μ represents the mean of the descriptor values and σ represents the standard deviation (Table S6).

**Figure 3:**
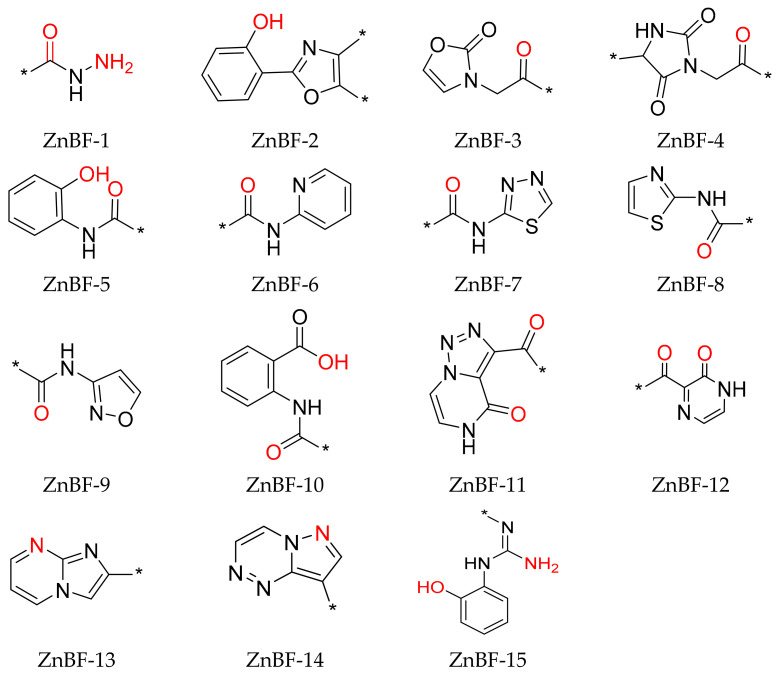
Representative ZnBFs identified by fragment library screening. Potential Zn^2+^-binding atoms are shown in red, and variable fragments are incorporated at the sites represented by *.

A total of 67 small-size molecules were selected, and the representative 15 molecules are shown in Figure S6. The corresponding ZnBFs presented in these molecules are listed in Figure 3 with the potential Zn^2+^-binding atoms shown in red. Heteroatoms of O/N/S/F within a distance of 2.8 Å from the metal atoms were selected as the binding atoms [22]. Among them, ZnBF-1, ZnBF-2, ZnBF-5, and ZnBF-6 are known Zn^2+^-binding fragments [25,26,27], which proves the reliability of the model. Novel ZnBFs can be recognized from fragment libraries with better structural novelty and diversity and can further guide the discovery of new ZnMIs.

### 2.6. Substructure Search and Virtual Screening for HDAC1 Inhibitors

To verify the feasibility of our strategy to identify inhibitors for any specific Zn^2+^-dependent metalloenzyme, the fifteen ZnBFs (Figure 3) were applied to screen the Specs library to obtain potential ZnMIs for HDAC1. Basically, a customized ZnBF pharmacophore feature [20,28] was generated by the Customize Pharmacophore Features protocol in DS 2018, which was defined by a ZnBF library containing the 15 ZnBFs listed in Figure 3. The customized ZnBF pharmacophore feature was set as a required feature and used for the substructure search in the Specs library for potential HDAC1 inhibitors.

**Figure 4:**
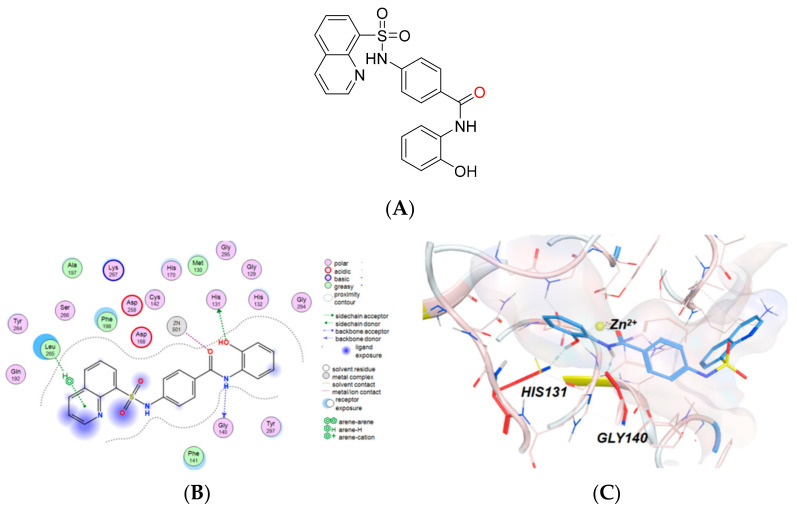
The structure (**A**) and interaction mode ((**B**): 2D diagram; (**C**): 3D diagram) of compound **9** with HDAC1.

The resulting hits were then docked with HDAC1, and ten molecules were prioritized for further biological testing. These compounds were initially tested for their percent inhibition against HDAC1 at the concentration of 100 μM. The structures and HDAC1 inhibitory activities of these compounds are shown in Table S7. Among them, compound **9** (Figure 4A) showed an inhibitory rate of 99.4% at 100 μM and was identified as a potent inhibitor against HDAC1. Further literature investigation showed that compound **9** was a known HDAC1 inhibitor with an IC_50_ of 0.957 μM [29]. The docking results (Figure 4B,C) indicated that compound **9** binds with the zinc ion through the amide oxygen in a monodentate manner. The amide nitrogen and the phenolic oxygen present hydrogen bonds with His131 and Gly140, respectively, which ensures the effective binding of the metal-binding fragment to the zinc ion.

The successful recognition of compound **9** without preliminary knowledge demonstrates the reliability of the screening strategy and encourages further application of this strategy to identify novel inhibitors for HDAC1 or other Zn^2+^-dependent metalloenzymes.

## 3. Materials and Methods

Molecular Operating Environment [30] (MOE) (2014.09) software, Discovery Studio 2018 [31] (DS 2018), and Padel [32] (http://www.yapcwsoft.com/dd/padeldescriptor/, accessed on 15 February 2024) software were used for descriptor calculation. The software package Weka [33] (3.8.5) (https://waikato.github.io/weka-wiki/, accessed on 15 February 2024) was used for descriptor selection and modeling. Glide [34] was used for molecular docking. The parameters were defined by default unless otherwise specified.

### 3.1. Properties Characterization of ZnMIs and Virtual Screening Strategy against HDAC1

**Figure 5:**
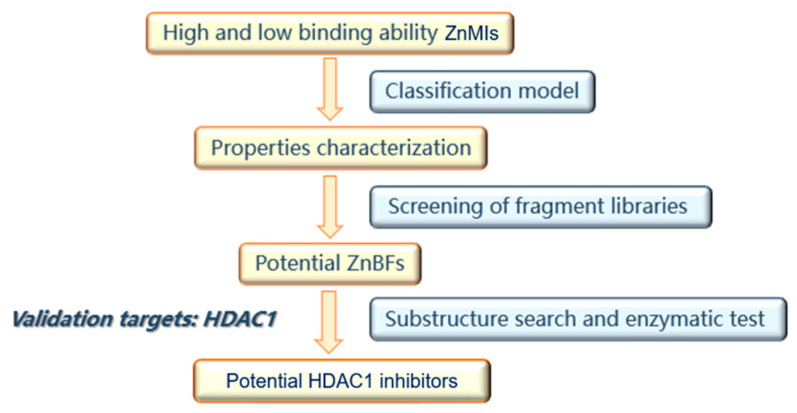
Property characterization-based strategy to discover novel inhibitors for HDAC1.

The primary goal of this work was to explore a generalized strategy based on property characterization to discover novel ZnMIs with unique ZnBFs. The strategy to identify novel ZnBFs and ZnMIs is shown in Figure 5. Since inhibitors targeting different metalloenzymes cannot be described by quantitative models, classification models were used in property characterization. Briefly, physicochemical, electronic, and DFT descriptors were generated for ligands with different binding affinities, and classification models were thus constructed. The electronical properties most relevant to the ZnBFs were revealed by analyzing the modeling descriptors. Both the classification models and the descriptor distribution ranges were used for fragment library screening to identify potential ZnBFs. ZnMIs were further obtained through ZnBF-based substructure searches. HDAC1 was used as the validation target, and the ZnMIs identified by this strategy were further selected through docking studies and evaluated with enzymatic tests against HDAC1.

### 3.2. Dataset Collection and Descriptor Selection

Chemical and enzymatic activity information regarding ZnMIs was collected from MetalPDB [35], Melad [22], Binding DB [36], Binding Moad [37], and PDB Bind [38] databases.

The DFT, physicochemical, and topological properties of the molecules in the data set were calculated by DS, MOE, and Padel, respectively. Four different methods were used for descriptor selection in WEKA, including Cfs subset evaluation (Cfs), gain ratio attribute evaluation (GR), wrapper subset evaluation (Wrapper), and Cfs combined with Wrapper (Cfs&wrapper). Among them, Cfs and GR are methods to select descriptors based on the predictive ability and gain ratio of a single descriptor for a specific category. Wrapper selects high-performance descriptor subsets through learning algorithms, such as Naive Bayesian, IBK, J48, etc. Naive Bayesian was used in this study.

### 3.3. Construction and Validation of the Classification Model

The interpretability of the model was emphasized in the selection of classification algorithms, and eight machine learning algorithms, including Naive Bayesian (NB), IBK, J48 [23], Attribute Selected Classifier (ASC)_NB, ASC_IBK, ASC_J48, ZeroR, and OneR, were adopted with WEKA. NB is a probability-based Bayesian classifier. IBK is a distance-based lazy classifier. J48 is a decision tree classifier based on descriptor information gain. ASC is a meta-classifier that takes the result of the classifier as the input of classification. In this study, the NB, IBK, and J48 classifiers were used in ASC, represented by ASC_NB, ASC_IBK, and ASC_J48, respectively. ZeroR and OneR are rule-based classifiers that use zero or one descriptor for model construction and were used herein to evaluate the rationality of attribute selection.

Predictive accuracy is primarily measured by classification accuracy defined by the following equation:

Classification Accuracy = number of correctly classified instances/total number of instances × 100%(1)

Parameters such as active _(detected)_% and hit rate (HR) were also used in model validation and were defined as follows:

Active _(detected)_ % = TP/(TP + FP) × 100% (2)

HR = TP/A × 100%(3)

where TP and FP were the number of true-positive and false-positive molecules found in the screened database, respectively, and A was the total number of active compounds in the entire database.

In addition, the ROC (Receiver Operating Characteristic) curve was used to evaluate the power of the model to differentiate between active and inactive compounds. The learning curve was also used to verify the reliability of our models.

### 3.4. Docking Method for HDAC1

Here, 1C3R [39] (PDB ID) was used as the receptor during docking with Glide. The distance threshold between the ligand and Zn^2+^ was defined as 2.5 Å, and the metal-binding interaction was defined as the required interaction. The ligand configuration was set to tetrahedral.

The effectiveness of the docking method was verified by docking its natural ligand TSN (the structure was shown in Figure S7) with the decoy set. The decoy set consisted of 1616 highly active HDAC1 inhibitors (IC_50_ < 100 nM), 101 inactive molecules collected from Chembl (IC_50_ > 100 μM), and 1500 putative inactive molecules selected from the ACD database with similar physical and chemical properties and different topological structures [40]. The RMSD value of the TSN re-docking conformation was 0.960 Å, which indicated good accuracy. When docked with decoy set molecules, the docking score of TSN (−9.037) was used as the cutoff value, and the active _(detected)_ % and HR were 81.9% and 37.5%, respectively. Furthermore, the AUC value of 0.744 indicated that the docking method has good discriminative power to distinguish active and inactive molecules.

### 3.5. Enzymatic Test against HDAC1

The enzymatic test against HDAC1 was performed by Chempartner (Shanghai, China). The compounds were tested in vitro using SAHA (purchased from Sigma -Aldrich, Merck KGaA, Darmstadt, Germany and/or its affiliates, Cat. No. SML0061) as the reference compound, and the testing concentration was set as 100 μM. HDAC1 was purchased from BPS bioscience (San Diego, CA, USA) and prepared in modified Tris Buffer. The substrate solution was made by trypsin and Ac-peptide substrate in modified Tris Buffer. A total of 15 μL of the HDAC1 solution was transferred to the assay plate and incubated at room temperature for 15 min; then, 10 μL of the substrate solution was added to each well to start the reaction. The fluorescence was then measured for excitation and emission at wavelengths of 355 nm and 460 nm by a Envision plate reader. The percentage inhibition was calculated using the following equation: Inhibition % = (Max-Signal)/(Max-Min) × 100%. All the tests were performed in duplicate.

## 4. Conclusions

To overcome poor specificity and drug-likeness related to currently available metal-binding fragments, chemoinformatic techniques were used to characterize ZnBFs and to discover new ZnMIs with improved property profiles. Fifteen potential ZnBFs were identified through property characterization, and virtual screening based on fragment recognition and molecular docking prioritized ten potential HDAC1 inhibitors for biological tests. Compound **9**, a known potent HDAC1 inhibitor, was successfully identified, which demonstrates the robustness of our strategy based on property characterization and virtual screening. Although ZnBF-5 is a known ZnBF and compound **9** is a known HDAC1 inhibitor, such a strategy is readily extendable. When applied to chemical libraries with better structural diversity and drug-likeness, it might recognize novel ZnMIs for specific Zn^2+^-dependent metalloenzymes.

## Figures and Tables

**Table 1:** Predictive accuracy (%) of models derived from different algorithms and descriptor selection methods.

Algorithms	Descriptor Selection Methods			
	Cfs	GR	Wrapper	Cfs&Wrapper
ASC_IBK	73.82	73.97	74.27	**75.60**
IBK	76.11	75.96	76.44	**76.50**
ASC_J48	72.36	**75.43**	71.49	73.97
J48	71.30	73.84	70.03	**74.12**
ASC_NB	76.09	**78.52**	77.60	76.85
NB	77.02	**82.82**	79.20	79.44
ZeroR	63.82	**63.82**	63.82	63.82
OneR	70.86	71.37	70.10	**72.01**

**Table 2:** Predictive accuracy (%) of the training, internal test, and external test sets for models built by ASC_NB or NB algorithms and different descriptor selection methods.

Algorithms		Descriptor Selection Methods			
		Cfs	GR	Wrapper	Cfs&Wrapper
ASC_NB	Training Set	75.32	72.17	81.25	79.25
	Internal Test Set	76.61	81.48	74.46	78.41
	External Test Set	70.32	69.76	72.09	71.14
NB	Training Set	77.49	76.52	77.67	**81.27**
	Internal Test Set	76.96	83.33	76.59	**82.27**
	External Test Set	73.01	69.76	69.76	**72.54**

**Table 3:** The AUC values of the ROC curves derived from models generated from training, internal test, and external test sets by the Cfs&Wrapper-NB method.

Model Names	AUC Values		
	Training Set	Internal Test Set	External Test Set
1	0.839	0.857	0.792
2	0.844	0.867	0.782
3	0.860	0.827	0.757
4	0.840	0.912	0.782
**5**	**0.860**	**0.822**	**0.801**
6	0.848	0.849	0.762
7	0.884	0.802	0.755
8	0.881	0.729	0.765
9	0.813	0.900	0.760
10	0.838	0.875	0.779

## Data Availability

Data are contained within the article and Supplementary Materials.

## Supplementary Materials

The following supporting information can be downloaded at: https://www.mdpi.com/article/10.3390/molecules29051096/s1, Figure S1: (a) The curve of IBK model prediction accuracy (%) changing with K values; (b) The curve of J48 model prediction accuracy (%) changing with Minumum Obj values; Figure S2: Structures of (a) training set, (b) internal test set, and (c) external test set; Figure S3: The false positive molecules predict by the model; Figure S4: The learning curve drawn by the NB method; Figure S5: (a)–(h) Distribution of JGI9, nAtomP, JGI8, HBA_Count, BCUTp-1h, PE-OE_VSA-4, PEOE_VSA+1, PE-OE_VSA-0 among different classes, respectively; Figure S6: Representative 15 fragment small molecules; Figure S7: Structure of TSN in crystal complex 1C3R; Table S1: Information of high frequency binding fragments in the high activity dataset of Zn^2+^ metalloenzymes; Table S2: Accuracy of models using IBK method with different K values; Table S3: Accuracy of models using J48 method with different Min Num Obj values; Table S4: Information of (a) training set, (b) internal test set and (c) external test set molecules; Table S5: Meanings and classification of the 34 descriptors using in Model 5. The sixteen highly relevant descriptors were shown in bold; Table S6: The descriptor range and interpretation of eight electronic descriptors related to MBFs in Model 5; Table S7: The structure and activity of 10 compounds obtained by virtual screening for HDAC1.
