# Supplementary material for: Discovery of Novel Metalloenzyme Inhibitors Based on Property Characterization: Strategy and Application for HDAC1 Inhibitors
Source: Molecules. 2024 Feb 29;29(5):1096. doi: 10.3390/molecules29051096 (PMC10935415; doi:10.3390/molecules29051096)
Supplement: Supplementary file 1 [file molecules-29-01096-s001.zip › molecules-2775757-supplementary.pdf]

Supplementary Material

Discovery of novel metalloenzyme inhibitors based on property characterization: Strategy and application for HDAC1 inhibitors

Lu Zhang[1,2], Yajun Yang[1], Ying Yang[1] and Zhiyan Xiao[1,3]*

1 Beijing Key Laboratory of Active Substance Discovery and Druggability Evaluation, Institute of Materia Medica, Chinese Academy of Medical Sciences and Peking Union Medical College, Beijing 100050, China.
2 Department of Toxicology, Tianjin Centers for Disease Control and Prevention, Tianjin, 300011, China.
3 State Key Laboratory of Digestive Health, Institute of Materia Medica, Chinese Academy of Medical
Sciences and Peking Union Medical College, Beijing 100050, China

*Correspondence: xiaoz@imm.ac.cn

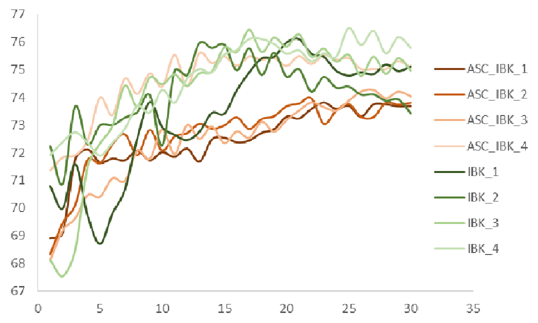

(a)

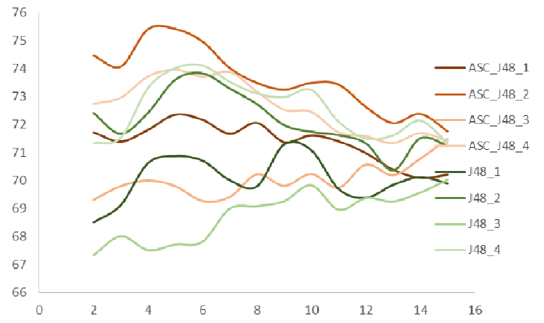

(b)

Figure S1. Predictive accuracy changing with the K value. **(a)** The curves derived from IBK models; **(b)** The curve derived from J48 models.

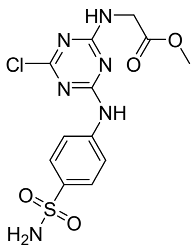

1

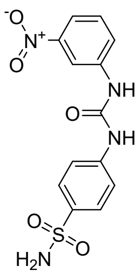

2

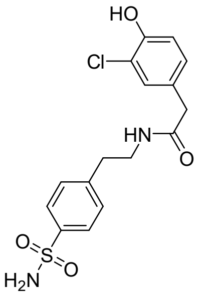

3

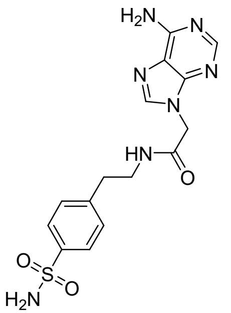

4

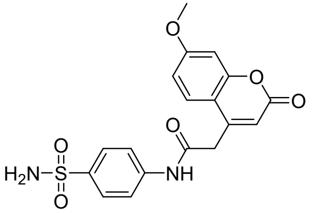

5

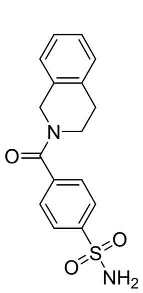

6

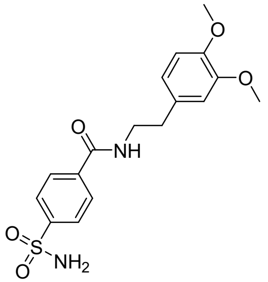

7

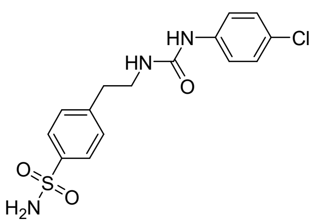

8

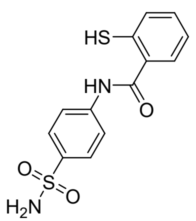

9

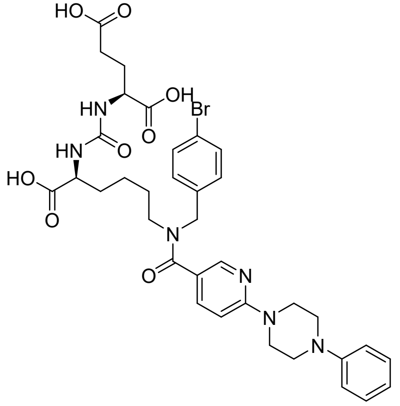

10

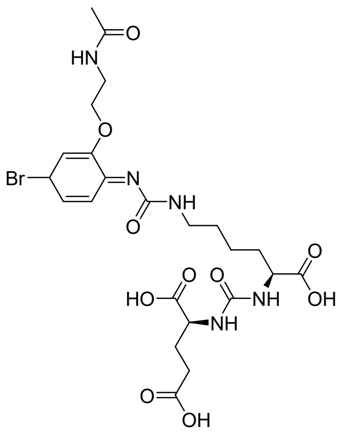

11

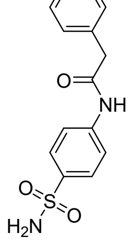

12

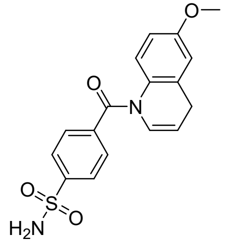

13

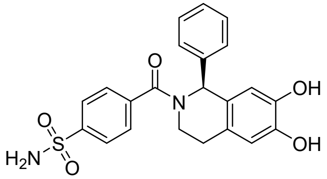

14

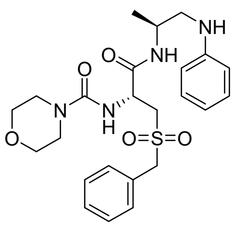

15

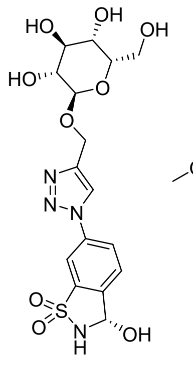

16

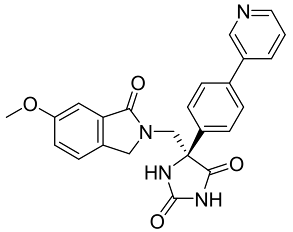

17

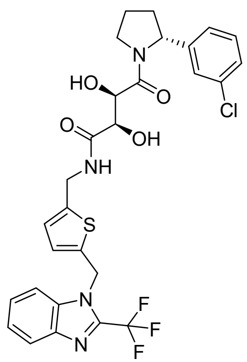

18

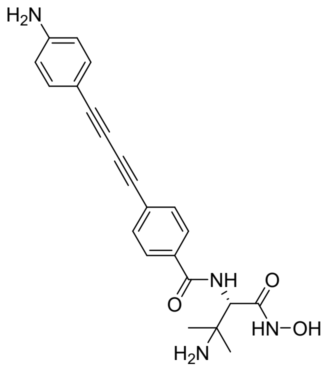

19

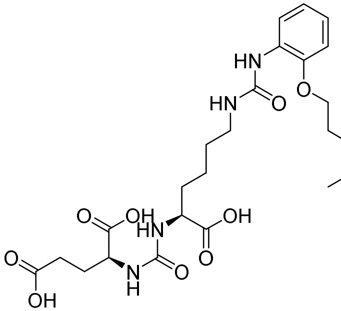

20

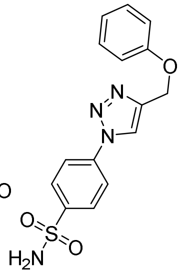

21

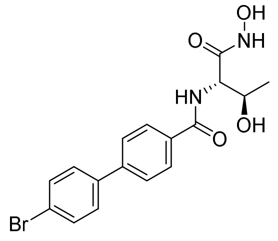

22

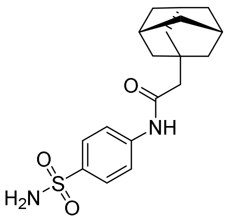

23

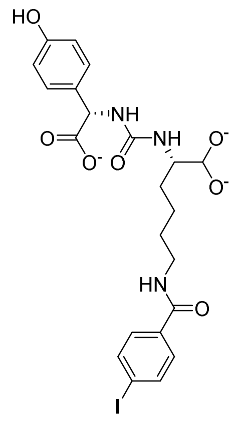

24

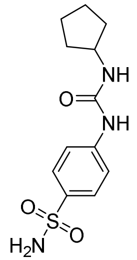

25

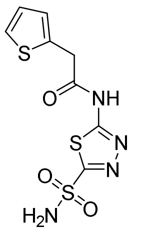

26

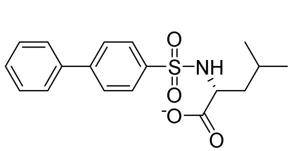

27

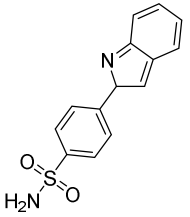

28

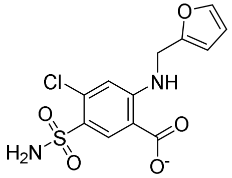

29

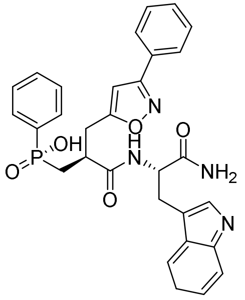

30

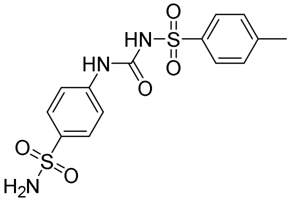

31

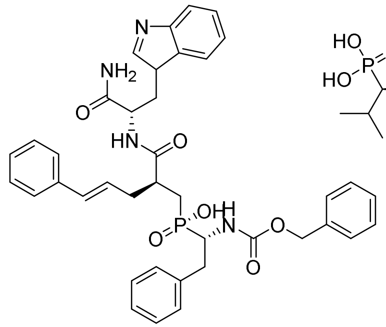

32

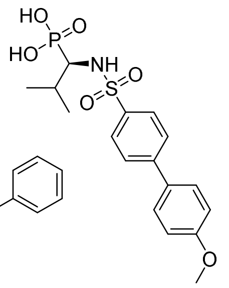

33

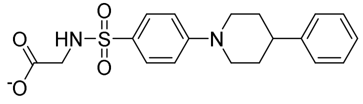

34

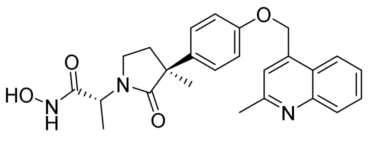

35

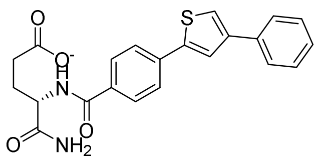

36

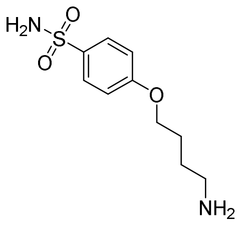

37

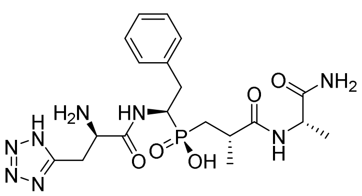

38

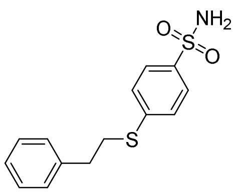

39

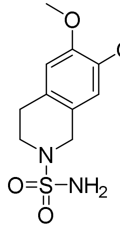

40

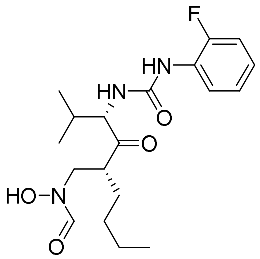

41

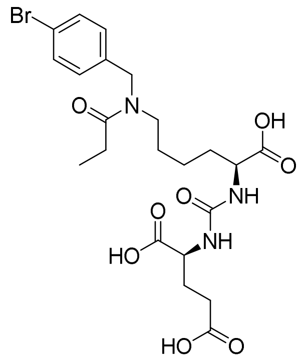

42

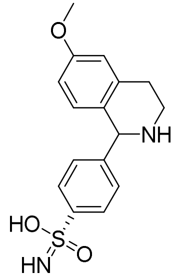

43

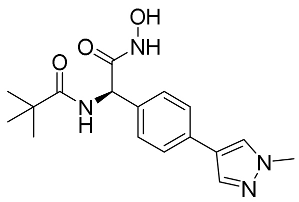

44

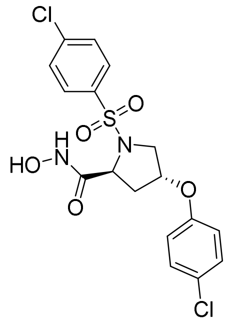

45

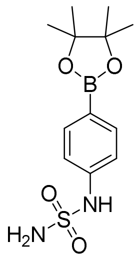

46

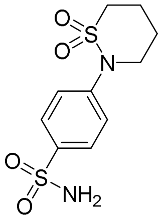

47

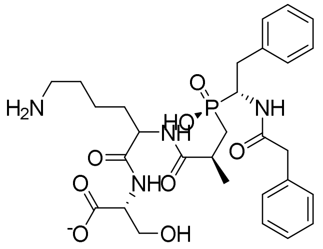

48

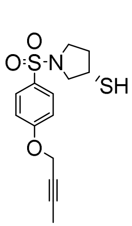

49

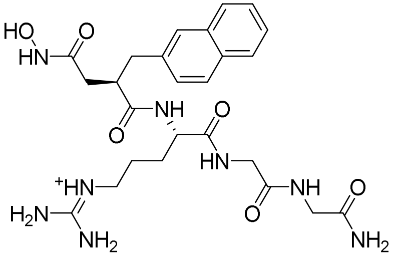

50

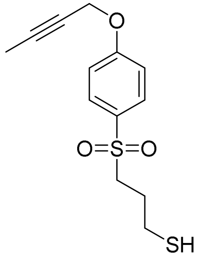

51

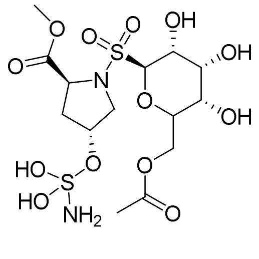

52

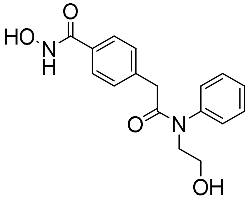

53

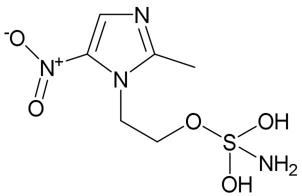

54

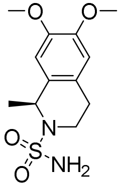

55

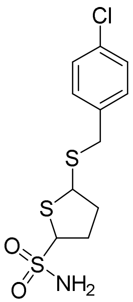

56

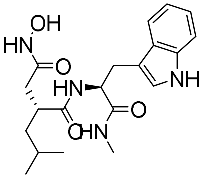

57

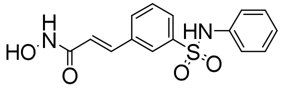

58

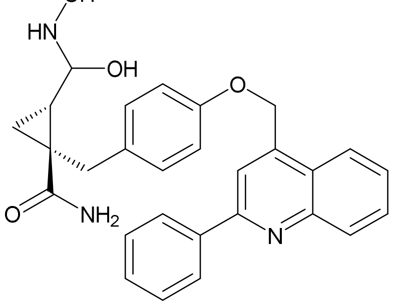

59

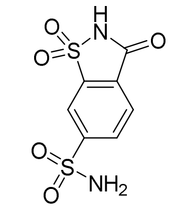

60

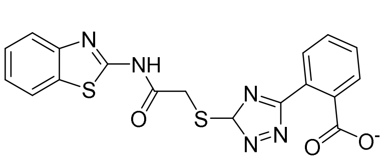

61

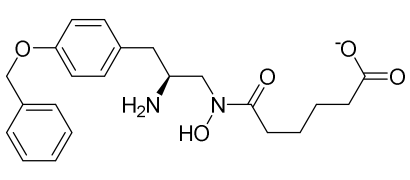

62

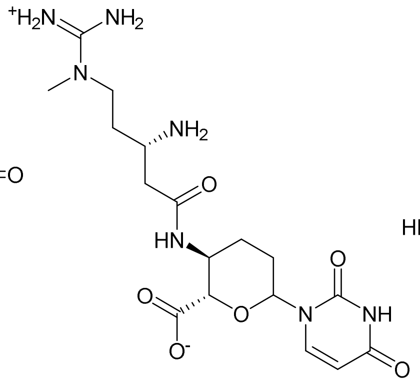

63

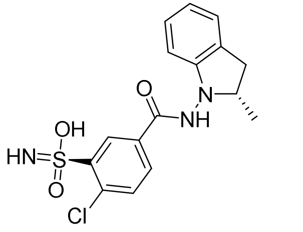

64

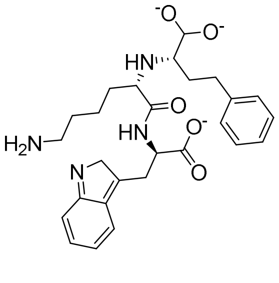

65

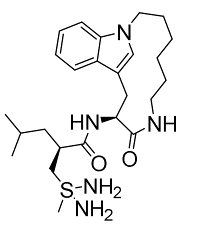

66

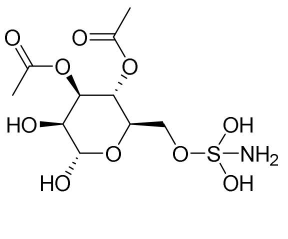

67

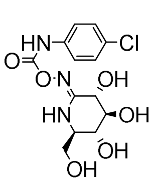

68

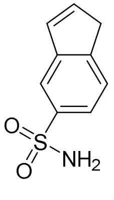

69

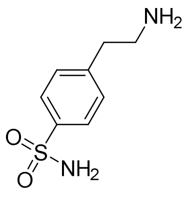

70

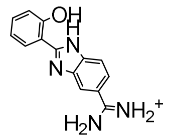

71

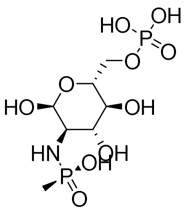

72

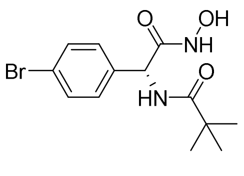

73

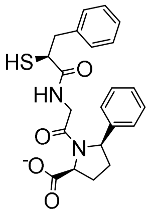

74

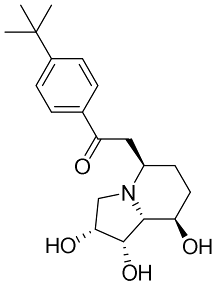

75

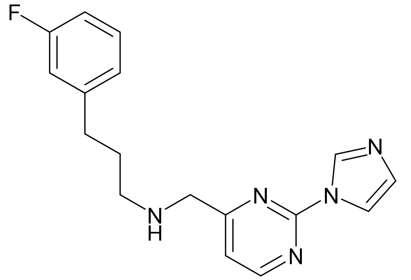

76

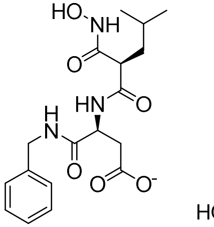

77

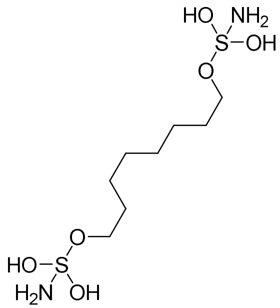

78

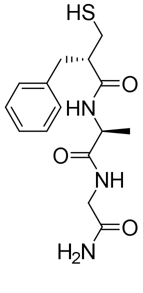

79

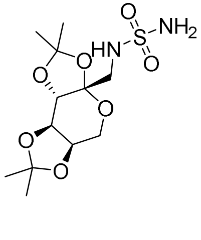

80

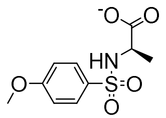

81

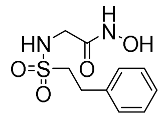

82

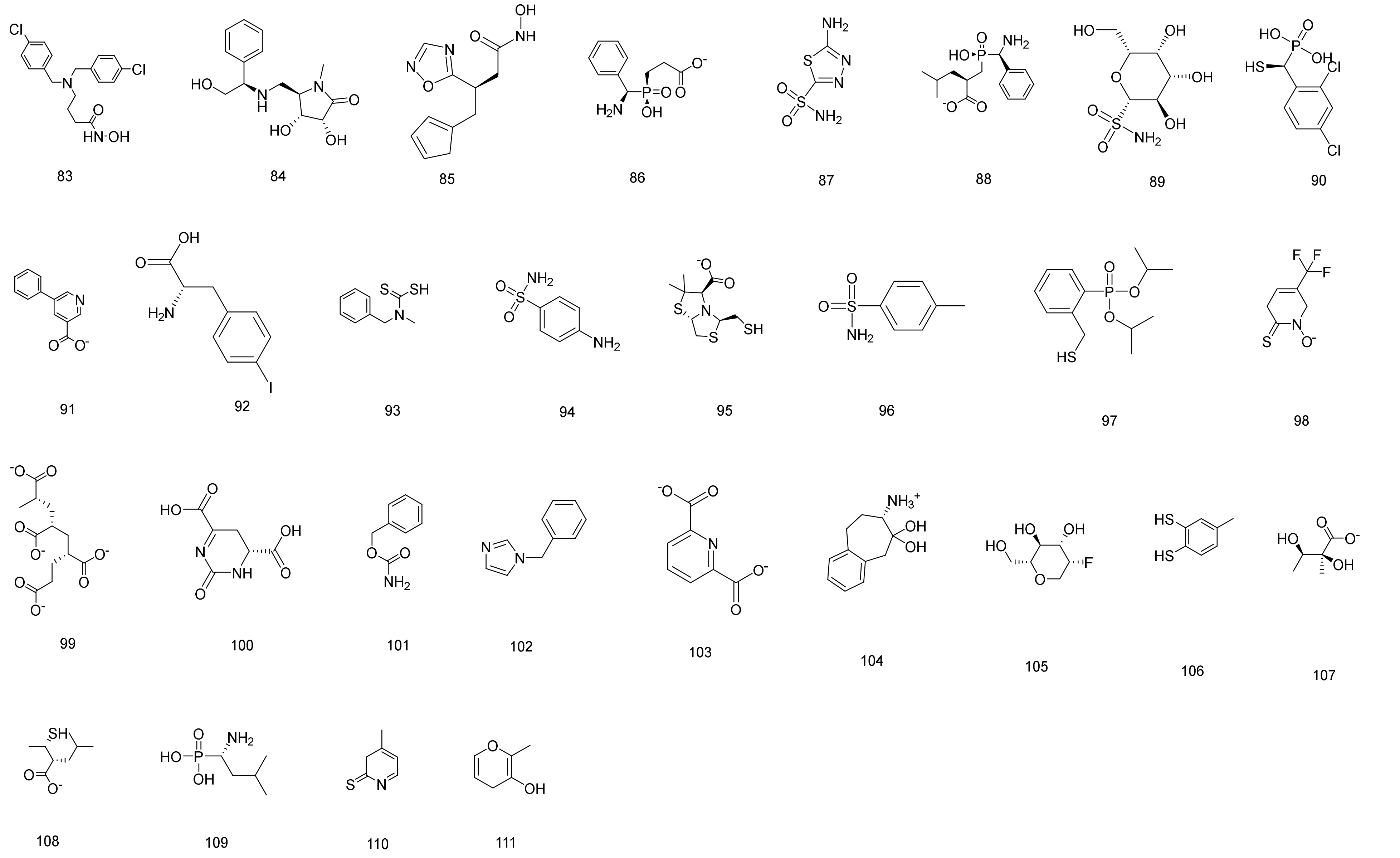
83
84
85
86
87
88
89
90
91
92
93
94
95
96
97
98
99
100
101
102
103
104
105
106
107
108
109
110
111

(a)

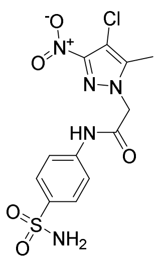

1

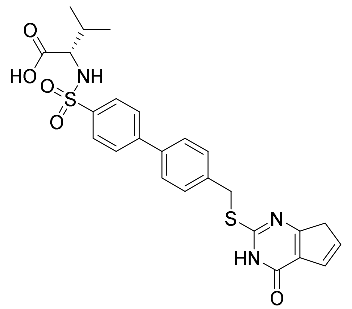

2

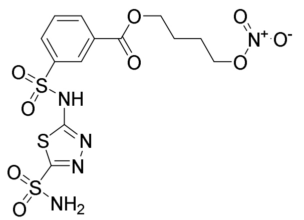

3

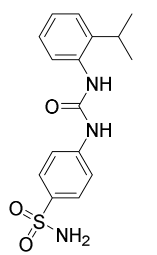

4

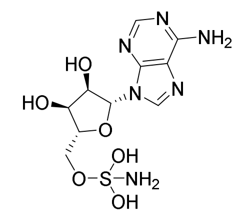

5

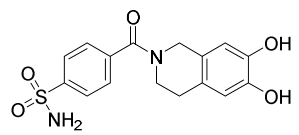

6

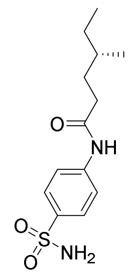

7

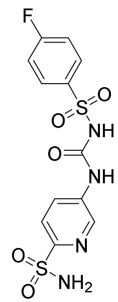

8

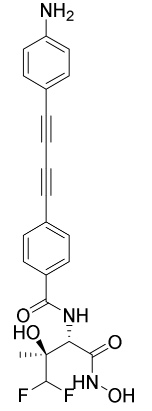

9

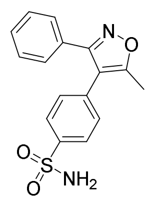

10

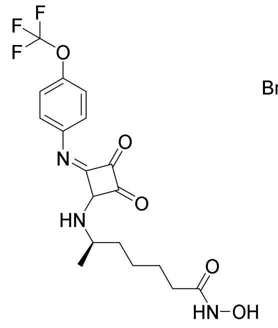

11

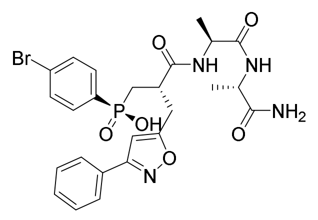

12

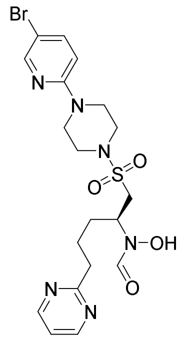

13

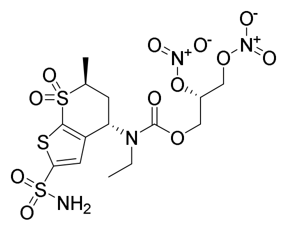

14

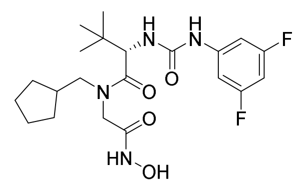

15

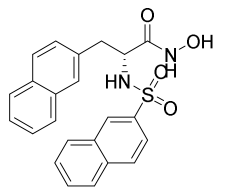

16

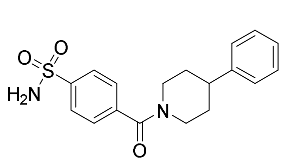

17

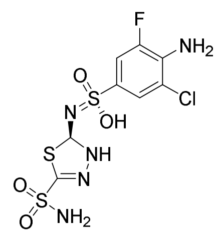

18

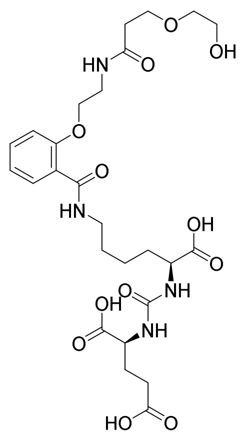

19

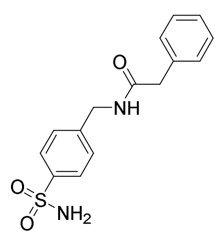

20

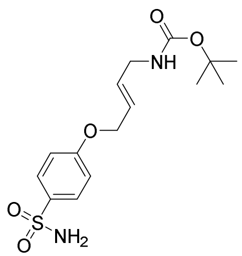

21

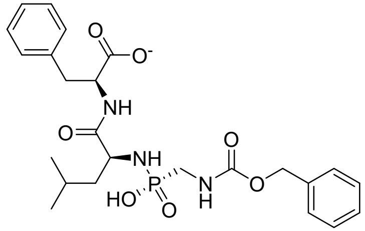

22

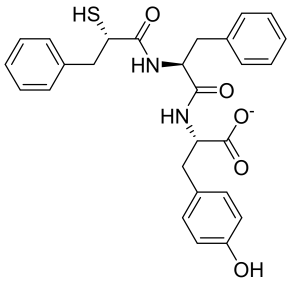

23

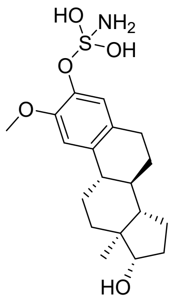

24

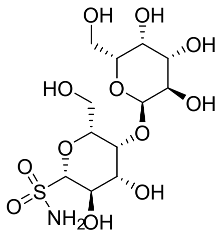

25

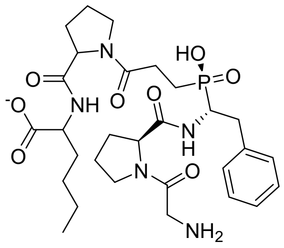

26

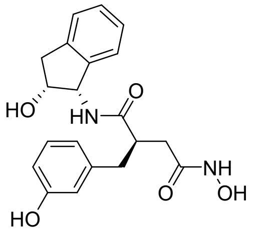

27

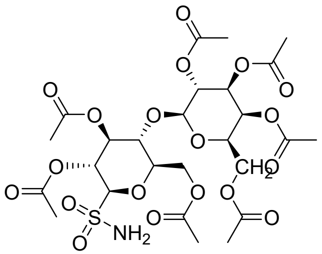

28

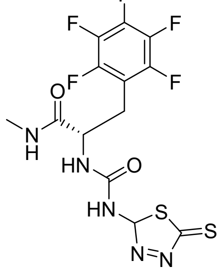

29

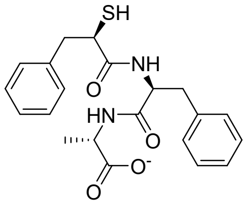

30

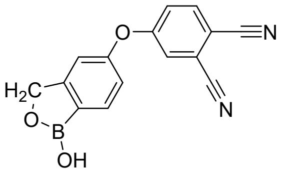

31

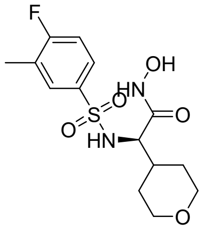

32

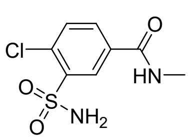

33

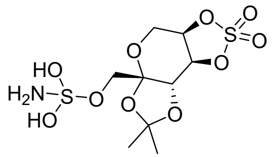

34

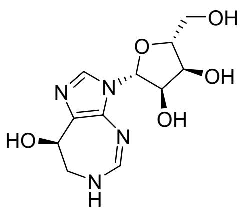

35

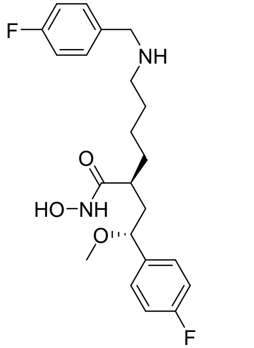

36

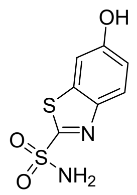

37

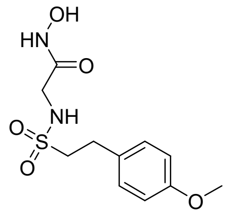

38

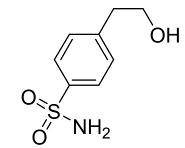

39

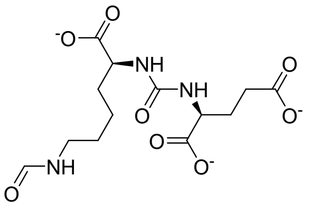

40

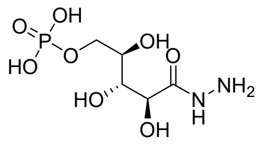

41

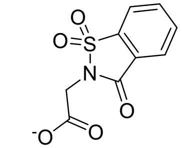

42

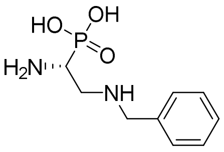

43

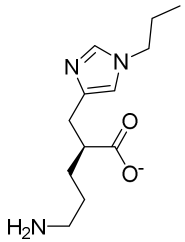

44

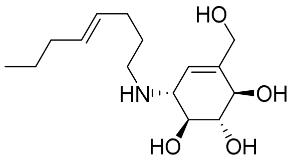

45

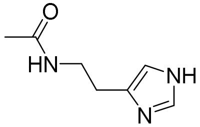

46

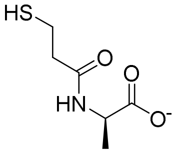

47

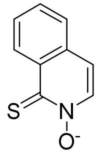

48

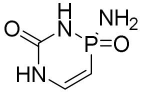

49

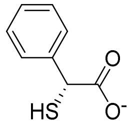

50

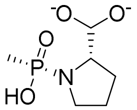

51

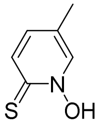

52

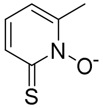

53

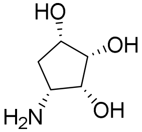

54

(b)

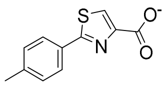

1

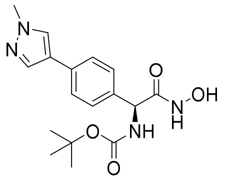

2

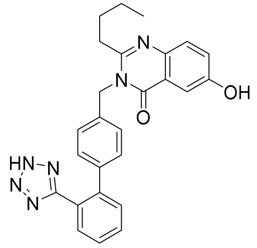

3

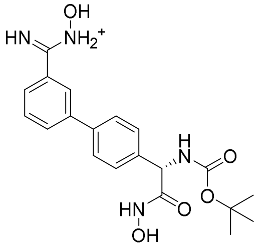

4

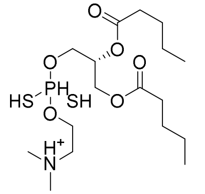

5

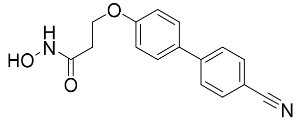

6

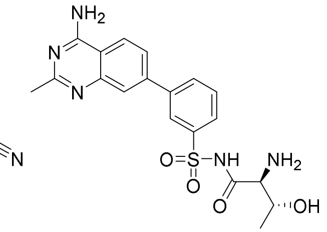

7

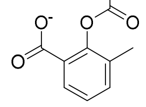

8

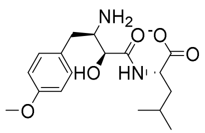

9

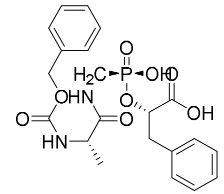

10

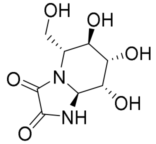

11

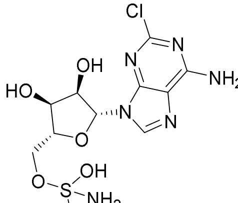

12

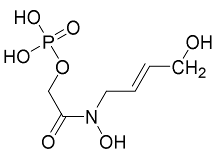

13

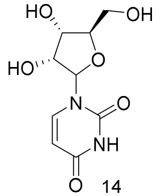

14

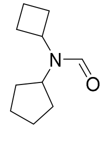

15

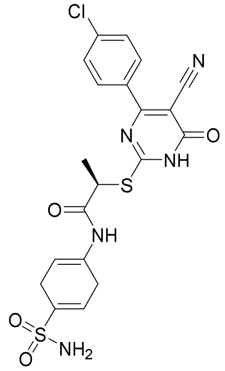

16

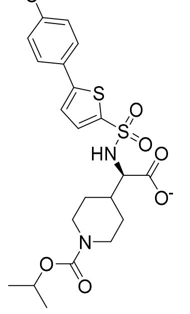

17

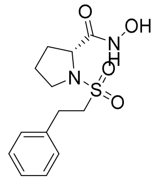

18

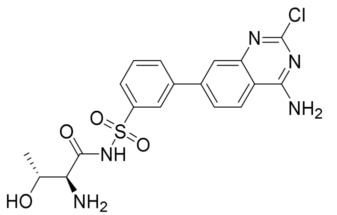

19

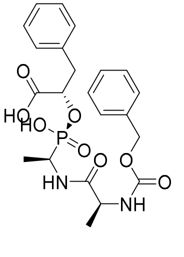

20

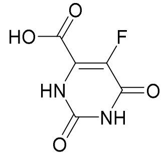

21

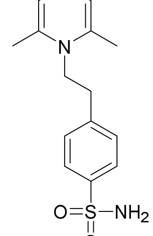

22

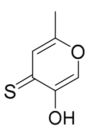

23

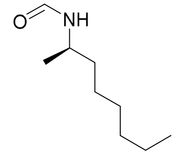

24

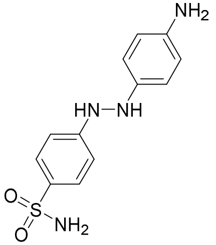

25

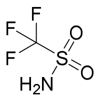

26

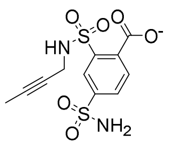

27

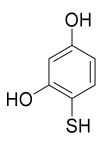

28

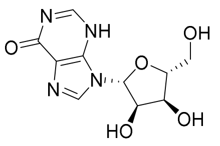

29

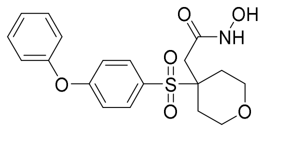

30

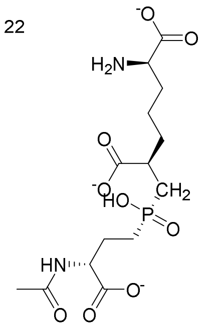

31

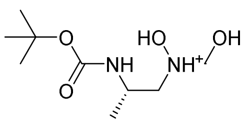

32

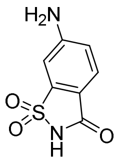

33

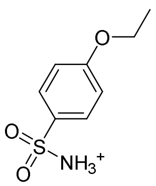

34

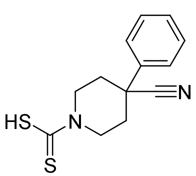

35

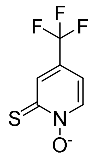

36

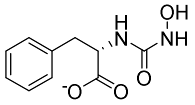

37

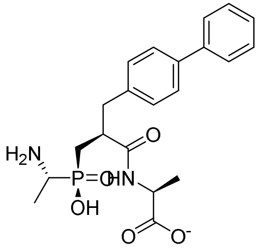

38

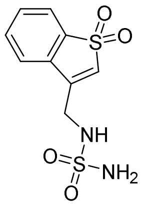

39

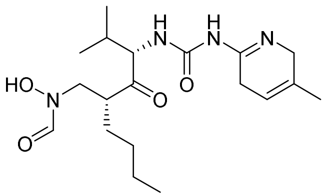

40

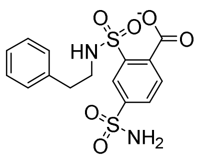

41

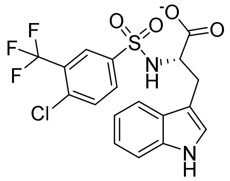

42

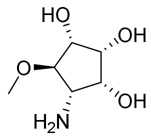

43

(c)

Figure S2. Structures of (a) training set, (b) internal test set, and (c) external test set molecules used for Model 5.

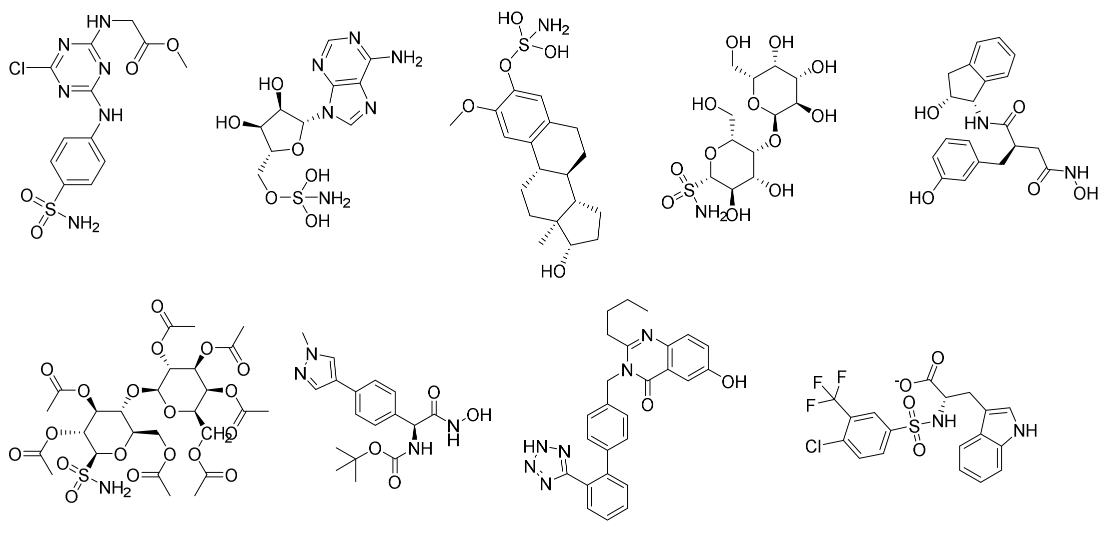

Figure S3. The false positive molecules predicted by Model 5.

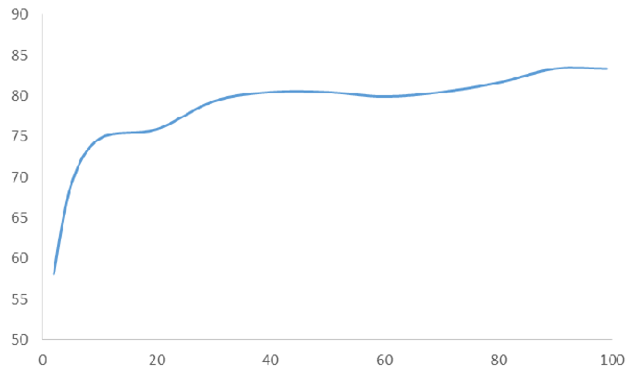

Figure S4. The learning curve derived from Model 5.

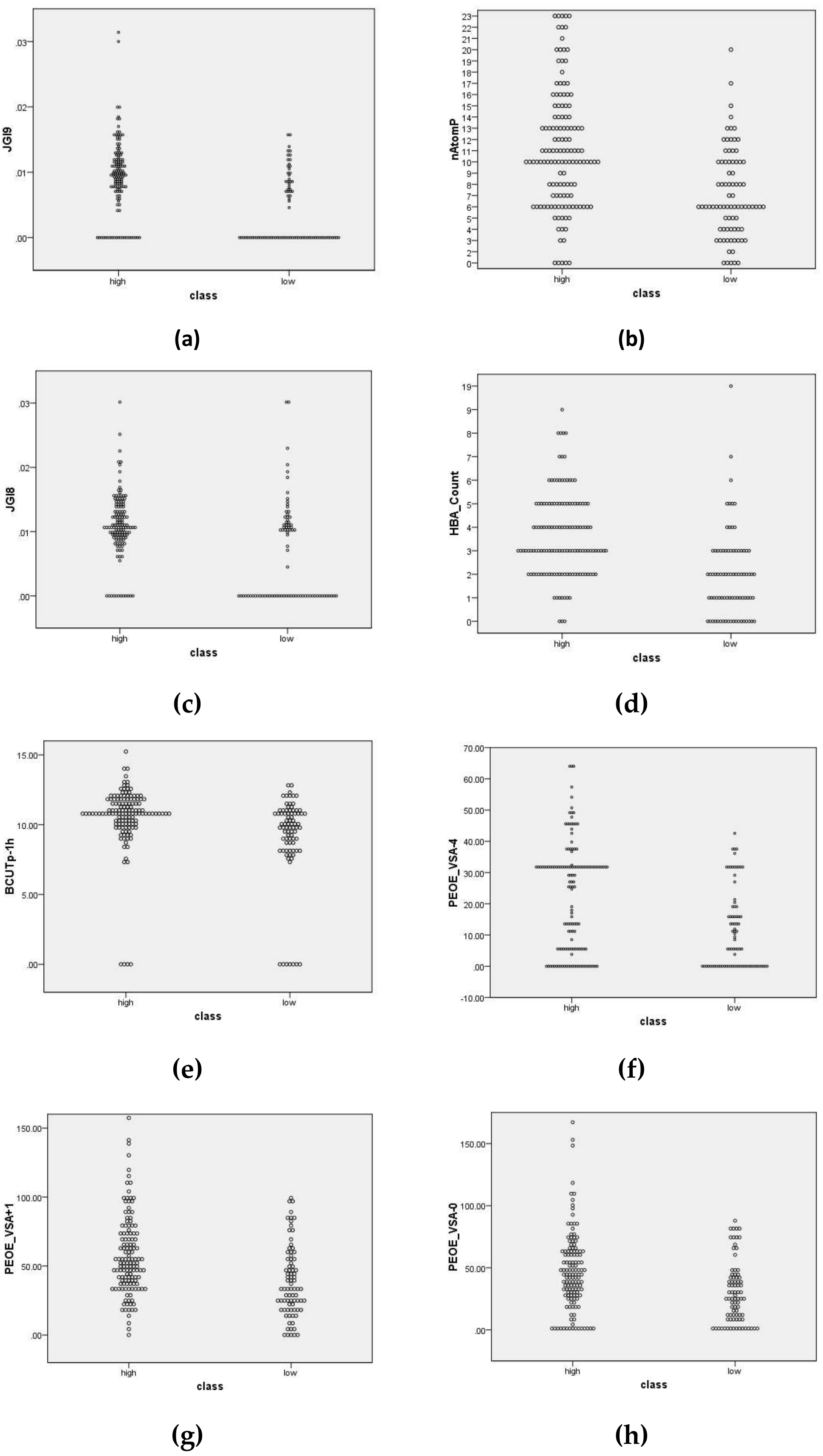

Figure S5. (a)-(h) Distribution of JGI9, nAtomP, JGI8, HBA_Count, BCUTp-1h, PE-OE_VSA-4, PEOE_VSA+1, PE-OE_VSA-0 among different classes, respectively.

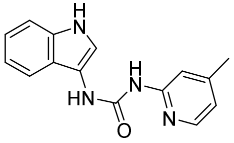
1

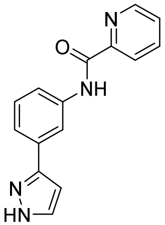
2

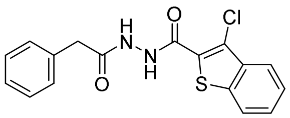
3

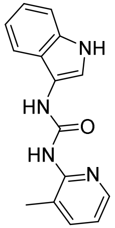
4

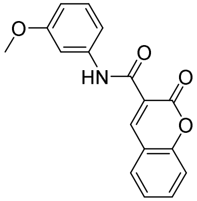
5

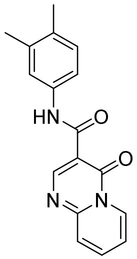
6

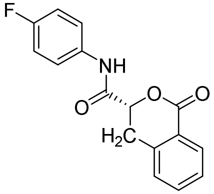
7

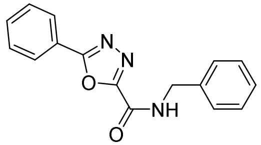
8

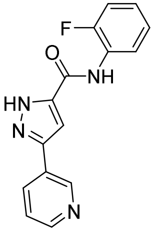
9

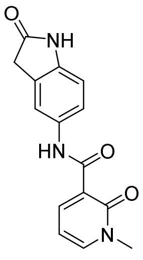
10

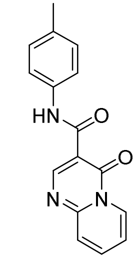
11

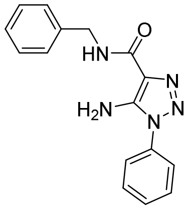
12

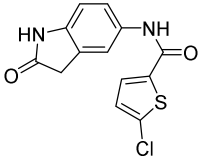
13

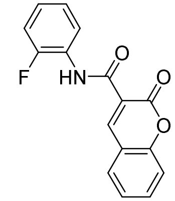
14

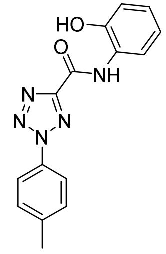
15

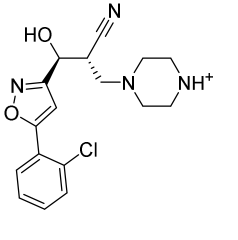
16

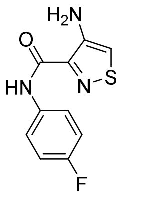
17

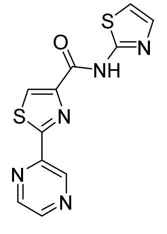
18

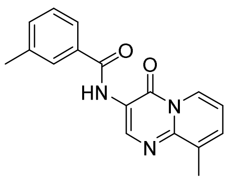
19

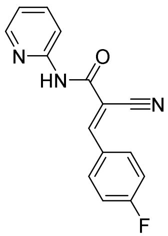
20

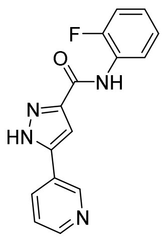
21

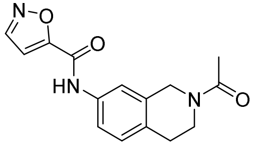
22

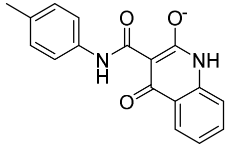
23

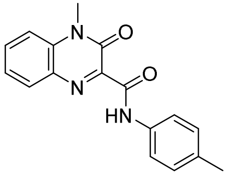
24

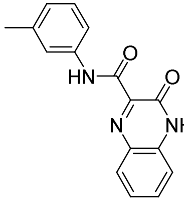
25

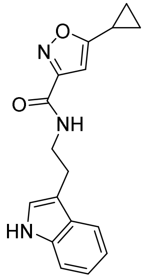
26

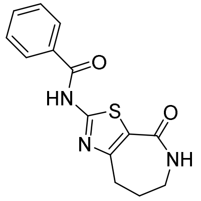
27

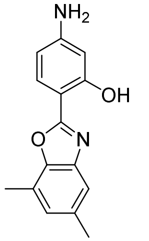
28

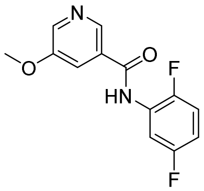
29

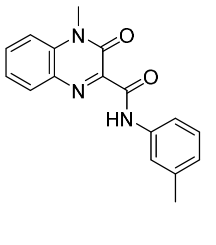
30

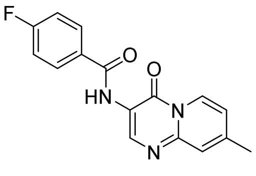
31

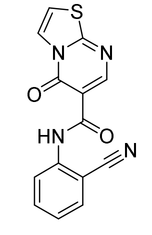
32

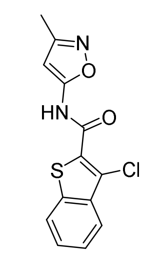

33

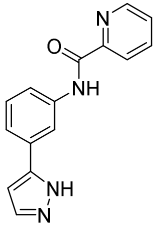

34

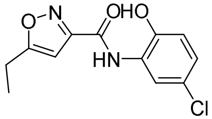

35

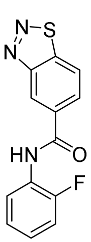

36

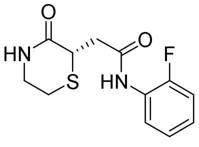

37

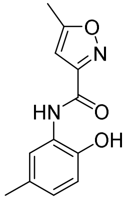

38

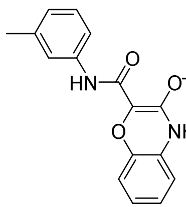

39

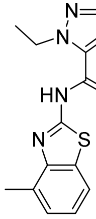

40

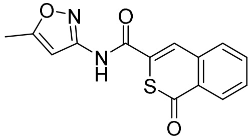

41

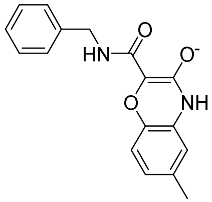

42

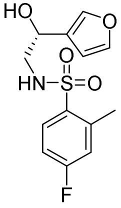

43

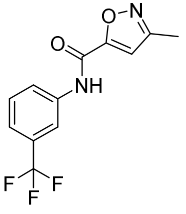

44

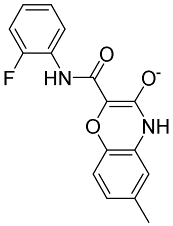

45

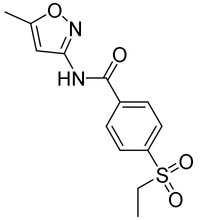

46

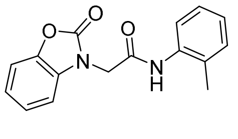

47

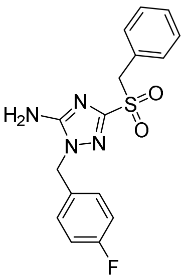

48

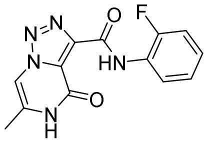

49

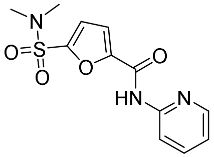

50

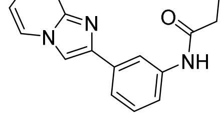

51

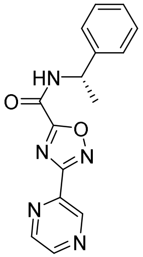

52

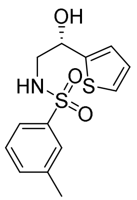

53

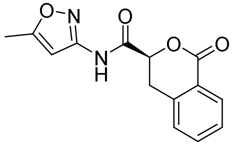

54

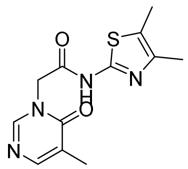

55

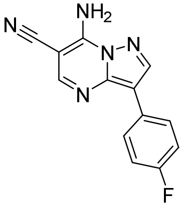

56

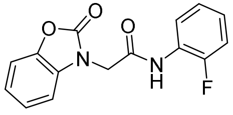

57

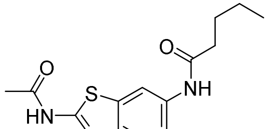

58

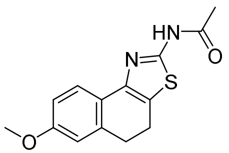

59

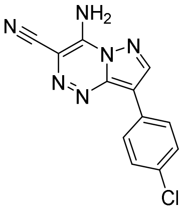

60

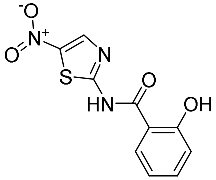

61

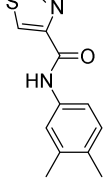

62

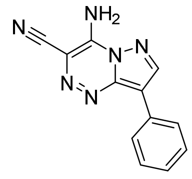

63

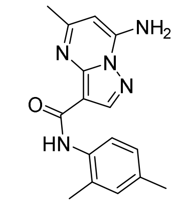

64

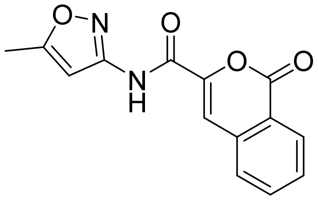

65

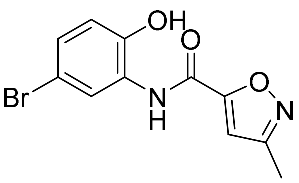

66

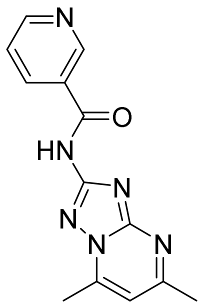

67

Figure S6. Structure of the selected 67 fragments.

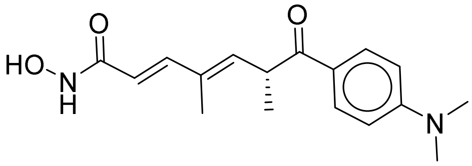

Figure S7. Structure of TSN in crystal complex 1C3R.

Table S1. Binding fragments with high frequency in the high activity data set of Zn^{2+}-dependent metalloenzymes.

MBP ID	Ligand	PDB ID	Metalloenzymes	Function	K_i (nM)
**MBP1-2-88**	F6W	6got	Carbonic Anhydrase 2	Lyase	0.40
	FKE	6h36	Carbonic Anhydrase 7	Lyase	0.45
	51J	5aml	Carbonic Anhydrase 2	Lyase	0.70
	8F3	5n0e	Carbonic Anhydrase 2	Lyase	0.83
	ANV	5ljq	Carbonic Anhydrase 2	Lyase	1.00
	WWV	3n3j	Carbonic Anhydrase 2	Lyase	1.28
	45I	5amd	Carbonic Anhydrase 2	Lyase	2.80
	5NXV	5nxv	Carbonic Anhydrase 2	Lyase	3.50
	D7A	3v7x	Carbonic Anhydrase 2	Lyase	4.85
	FC4	5j8z	Carbonic Anhydrase 2	Lyase	5.00
	4KB	4z1k	Carbonic Anhydrase 2	Lyase	5.22
	DF5	4z1e	Carbonic Anhydrase 2	Lyase	5.70
	3O1	4rfc	Carbonic Anhydrase 2	Lyase	6.13
	OSP	2q1q	Carbonic Anhydrase 2	Lyase	7.00
	4KC	4z1j	Carbonic Anhydrase 2	Lyase	7.60
	CQS	6b59	Carbonic Anhydrase 2	Lyase	8.20
	4RFD	4rfd	Carbonic Anhydrase 2	Lyase	8.90
	1ZFK	1zfk	Carbonic Anhydrase 2	Lyase	9.17
	1EZ	4ilx	Carbonic Anhydrase 2	Lyase	9.40
	OYQ	3oyq	Carbonic Anhydrase 2	Lyase	10.30
	3EFT	3eft	Carbonic Anhydrase 2	Lyase	12.00
	9EB	5ny6	Carbonic Anhydrase 2	Lyase	13.50

	AYX	3n2p	Carbonic Anhydrase 2	Lyase	15.00
	DWH	3mna	Carbonic Anhydrase 2	Lyase	16.32
	R21	3nb5	Carbonic Anhydrase 2	Lyase	18.00
	PIU	1ze8	Carbonic Anhydrase 2	Lyase	21.00
	4RUZ	4ruz	Carbonic Anhydrase 2	Lyase	23.10
	SU0	3ml2	Carbonic Anhydrase 2	Lyase	25.63
	4WA	5byi	Carbonic Anhydrase 2	Lyase	30.70
	OYS	3oys	Carbonic Anhydrase 2	Lyase	31.18
	9E+08	5ny3	Carbonic Anhydrase 2	Lyase	33.90
	S6I	3mzc	Carbonic Anhydrase 2	Lyase	40.62
	COX	2aw1	Carbonic Anhydrase 2	Lyase	43.00
	1GD	4itp	Carbonic Anhydrase 2	Lyase	63.64
	FUN	1z9y	Carbonic Anhydrase 2	Lyase	65.00
	BOS	2hd6	Carbonic Anhydrase 2	Lyase	66.45
	0FZ	3vbd	Carbonic Anhydrase 2	Lyase	76.53
	8GE	5n1s	Carbonic Anhydrase 2	Lyase	81.80
	5EN	5drr	UDP-3-O-acyl-N-acetylglucosamine Deacetylase lpxC	Hydrolase	0.00
	5EM	5drq	UDP-3-O-acyl-N-acetylglucosamine Deacetylase lpxC	Hydrolase	0.01
	541	2fv5	ADAM 17 Endopeptidase	Hydrolase	0.06
	615	3e8r	ADAM 17 Endopeptidase	Hydrolase	0.18
	0LX	4dv8	Anthrax Lethal Factor Endopeptidase	Hydrolase	0.58
	EL8	6g3o	Histone Deacetylase 2	Hydrolase	0.58
	L58	4fw5	UDP-3-O-acyl-N-acetylglucosamine Deacetylase lpxC	Hydrolase	1.00
**MBP2-1-9**	5OG	5een	Histone Deacetylase	Hydrolase	1.10

	QIX	3e4a	Insulin-Degrading Enzyme	Hydrolase	1.70
	4U5	4zy1	M17 Family Aminopeptidase	Hydrolase	7.20
	KLJ	4guy	Macrophage Metalloelastase	Hydrolase	10.00
	GVR	2ves	UDP-3-O-acyl-N-acetylglucosamine Deacetylase lpxC	Hydrolase	20.00
	RS2	966c	Fibroblast Collagenase	Hydrolase	23.00
	915	1yqy	Anthrax Lethal Factor Endopeptidase	Hydrolase	24.00
	JT6	2jt6	Stromelysin 1	Hydrolase	25.00
	7JT	5jf5	Peptide Deformylase	Hydrolase	63.00
	4TK	4zx3	M1 Family Aminopeptidase	Hydrolase	65.00
**MBP1-1-38**	ZAF	6cpa	Carboxypeptidase A1	Hydrolase	0.00
	BIR	1r1h	Neprilysin	Hydrolase	1.20
	BHX	3bhx	Glutamate Carboxypeptidase 2	Hydrolase	1.90
	1IU	4bxk	Peptidyl-Dipeptidase A	Hydrolase	11.21
	5MR	2ovz	Matrix Metalloproteinase-9	Hydrolase	13.00
	RXP	1hv5	Stromelysin 3	Hydrolase	22.91
	3A2	3aho	Oligopeptidase	Hydrolase	88.90
	3A1	3ahn	Oligopeptidase	Hydrolase	90.10
**MBP1-1-12**	686	4x3r	Glutamate Carboxypeptidase 2	Hydrolase	0.01
	J37	4ngq	Glutamate Carboxypeptidase 2	Hydrolase	1.57
	J42	4ngt	Glutamate Carboxypeptidase 2	Hydrolase	3.89
	J34	4ngs	Glutamate Carboxypeptidase 2	Hydrolase	7.60
	J31	4ngp	Glutamate Carboxypeptidase 2	Hydrolase	10.99
	J96	4ngn	Glutamate Carboxypeptidase 2	Hydrolase	28.10
	2QM	4oc5	Glutamate Carboxypeptidase 2	Hydrolase	85.05
**MBP1-2-81**	5L2	4zx0	Carbonic Anhydrase 2	Lyase	10.00
	SG7	3t85	Carbonic Anhydrase 2	Lyase	11.30

1VQ	5o07	Carbonic Anhydrase 2	Lyase	33.90
SMS	1eou	Carbonic Anhydrase 2	Lyase	36.00
O59	3ibl	Carbonic Anhydrase 2	Lyase	74.29
EO7	6c7x	Carbonic Anhydrase 2	Lyase	96.56

Table S2. Predictive accuracy of models using IBK method with different K values.

K value	AttributeSelectedClassifier_IBK				IBK			
	1	2	3	4	1	2	3	4
1	68.92	68.34	68.13	71.36	70.79	72.24	68.12	71.92
2	69.11	69.47	69.2	71.83	69.99	70.88	67.54	72.39
3	71.76	70.11	69.63	71.91	71.59	73.67	68.48	72.74
4	72.12	71.75	70.47	72.44	69.74	72.3	71.48	72.36
5	71.65	71.62	70.41	73.99	68.71	72.99	72.33	71.9
6	71.79	72.31	71.08	73.36	69.81	73	72.98	72.36
7	71.73	72.67	70.98	74.68	70.66	73.27	74.42	72.87
8	72.08	71.91	72.08	74.12	72.52	73.47	73.68	73.69
9	71.73	72.82	71.78	74.86	73.83	74.09	74.72	73.46
10	72.02	72.08	72.84	74.39	72.93	72.26	74.47	74.27
11	71.85	72.59	71.95	75.54	72.65	74.96	74.83	73.81
12	72.15	72.71	73	74.39	72.46	74.8	74.42	74.68
13	71.68	73.05	72.5	**75.6**	72.76	**75.96**	74.87	75.02
14	72.48	72.87	72.95	75.24	73.44	75.78	74.94	74.9
15	72.54	72.99	72.35	75.48	73.41	75.9	75.8	75.54
16	72.38	73.28	72.77	75.14	74.26	74.98	75.64	75.71
17	72.44	72.86	72.54	75.48	74.9	75.77	**76.44**	76.11

18	72.72	73.22	73.12	75.37	75.41	74.81	75.64	76.11
19	72.85	73.32	72.76	75.43	75.47	75.61	76.16	75.93
20	73.3	73.68	73.18	75.13	75.98	74.75	75.83	75.58
21	73.24	73.78	73.52	75.49	**76.11**	75.02	76.28	75.7
22	73.58	**73.97**	73.82	75.2	75.58	74.21	75.47	75.3
23	**73.82**	73.05	73.65	75.54	75.47	74.74	75.76	75.59
24	73.58	73.52	73.52	75.37	74.96	74.35	75.29	75.47
25	73.7	73.76	73.88	75.42	74.79	74.39	75.54	**76.5**
26	73.3	73.3	74.21	75.02	74.9	74.1	74.78	75.89
27	73.75	73.29	**74.27**	75.03	74.84	74.11	75.47	76.4
28	73.75	73.81	73.97	75.02	75.19	73.88	74.85	75.59
29	73.68	73.74	74.21	75.31	74.95	73.93	75.42	76.18
30	73.69	73.81	74.04	75.07	75.12	73.42	74.96	75.78
max	73.82	73.97	74.27	**75.6**	76.11	75.96	76.44	**76.5**

Table S3. Predictive accuracy of models using J48 method with different Min Num Obj values.

Min Num	AttributeSelectedClassifier_J48				J48			
Obj value	1	2	3	4	1	2	3	4
2	71.73	74.48	69.32	72.75	68.51	72.42	67.34	71.34
3	71.4	74.08	69.81	72.97	69.13	71.68	68.02	71.54
4	71.82	75.42	70.01	73.73	70.63	72.43	67.52	73.32
5	**72.36**	**75.43**	69.81	**73.97**	70.88	73.61	67.72	74.02
6	72.18	74.98	69.28	73.72	70.72	**73.84**	67.82	**74.12**
7	71.68	74.03	69.42	73.89	70.02	73.3	69	73.54
8	72.07	73.5	70.23	73.19	69.8	72.74	69.09	73.12
9	71.38	73.26	69.82	72.54	**71.3**	71.99	69.27	72.99
10	71.62	73.5	70.24	72.46	71.09	71.75	69.84	73.25
11	71.41	73.44	69.75	71.73	69.71	71.63	68.96	72.11
12	70.99	72.64	70.58	71.59	69.39	71.34	69.38	71.51
13	70.4	72.06	70.2	71.35	69.85	70.37	69.26	71.62
14	70.1	72.39	70.79	71.7	70.13	71.52	69.58	72.15
15	70.22	71.77	**71.49**	71.42	69.9	71.24	**70.03**	71.33
max	72.36	**75.43**	71.49	73.97	71.3	73.84	70.03	74.12

Table S4. Information of (a) training set, (b) internal test set and (c) external test set molecules.

(a)

No.	Ligand name	PDB ID	Ki (nM)	Class	No.	Ligand name	PDB ID	Ki (nM)	Class
1	DWH	3mna	16.32	high	2	AYX	3n2p	15.00	high
3	R21	3nb5	18.00	high	4	CQS	6b59	8.20	high
5	SU0	3ml2	25.63	high	6	4KC	4z1j	7.60	high
7	D7A	3v7x	4.85	high	8	9E08	5ny3	33.90	high
9	BOS	2hd6	66.45	high	10	686	4x3r	0.01	high
11	J42	4ngt	3.89	high	12	OYS	3oys	31.18	high
13	DF5	4z1e	5.70	high	14	8F3	5n0e	0.83	high
15	GNQ	2hh5	32.00	high	16	3QR	4riu	50.00	high
17	Z93	3lea	0.80	high	18	3LGP	3lgp	2.00	high
19	5EM	5drq	0.01	high	20	J34	4ngs	7.60	high
21	ANV	5ljq	1.00	high	22	L58	4fw5	1.00	high
23	1EZ	4ilx	9.40	high	24	2QM	4oc5	85.05	high
25	S6I	3mzc	40.62	high	26	1GO	4iwz	60.60	high
27	BDL	3ehx	25.00	high	28	8GE	5n1s	81.80	high
29	FUN	1z9y	65.00	high	30	5MR	2ovz	13.00	high
31	1ZFK	1zfk	9.17	high	32	RXP	1hv5	22.91	high
33	FIN	1zvx	1.73	high	34	IN7	1b8y	14.00	high

35	541	2fv5	0.06	high	36	E37	4efs	17.82	high
37	4RFD	4rfd	8.90	high	38	1IU	4bxk	11.21	high
39	F6W	6got	0.40	high	40	3IGP	3igp	22.59	high
41	FHF	3u7m	35.10	high	42	J37	4ngq	1.57	high
43	0FZ	3vbd	76.53	high	44	4U5	4zy1	7.20	high
45	QI3	3qj0	6300.00	low	46	BON	3mnu	66.45	high
47	OSP	2q1q	7.00	high	48	3A1	3ahn	90.10	high
49	283	2oi0	30.40	high	50	QIX	3e4a	1.70	high
51	440	3b92	10.00	high	52	5L2	4zx0	10.00	high
53	5OJ	5ef7	1.00	high	54	1VQ	5o07	33.90	high
55	DT9	3po6	23.64	high	56	7O8	5mjn	2.90	high
57	GM6	1pwu	2100.00	low	58	5OG	5een	1.10	high
59	615	3e8r	0.18	high	60	IL5	4xe1	77.30	high
61	752	5lsc	1200.00	low	62	HA2	2vj8	2.00	high
63	BLO	2z3h	23000.00	low	64	3BL1	3bl1	11436.30	low
65	X95	2x95	11300.00	low	66	SRS	1mmr	4000.00	low
67	SG7	3t85	11.30	high	68	GHR	3d52	520000.	low

								00	
69	2QOA	2qoa	52.00	high	70	ZYX	2nng	21000.00	low
71	122	1gi4	65.00	high	72	NNG	2p53	35.00	high
73	4TK	4zx3	65.00	high	74	TI3	1qf2	1200.00	low
75	HN4	3ejr	2.70	high	76	S8F	4v3v	2700.00	low
77	0ZB	1a86	88000.00	low	78	O59	3ibl	74.29	high
79	0D3	1jao	1200.00	low	80	B19	2h15	2713.99	low
81	TBL	3ehy	1400.00	low	82	KLG	3rts	10700.00	low
83	QI1	3qiy	4600.00	low	84	GB7	3ddg	1000.00	low
85	7JT	5jf5	63.00	high	86	4ZN	5cbm	17.00	high
87	1SA	2hnc	60.00	high	88	4QP	4zqt	2300.00	low
89	1SD	3hkq	4550.00	low	90	SDF	3iog	1095.45	low
91	0LO	4dsy	37000.00	low	92	PHI	1tf9	920000.00	low
93	P58	3p58	25.40	high	94	4NZ	5wcm	100000.00	low
95	9BZ	5ewa	7549.83	low	96	6LH	5jnc	25000.00	low
97	IFS	3iof	9165.15	low	98	7FH	4q8x	270000.	low

								00	
99	PAY	2v77	8700.00	low	100	OTD	2eg7	1300.00	low
101	FK8	6h29	2400.00	low	102	1BN	2afx	7100.00	low
103	PDC	4ih3	15200.00	low	104	5MFR	5mfr	50000.00	low
105	FMF	1qx1	7500000.00	low	106	TLD	2z94	1400.00	low
107	23B	4bt5	1720000.00	low	108	O2S	5om9	56000.00	low
109	2WW	4pvb	249000.00	low	110	4H2	4q90	7000000.00	low
111	TM4	4mlt	1400000.00	low					

(b)

No.	Ligand name	PDB ID	Ki (nM)	Class	No.	Ligand name	PDB ID	Ki (nM)	Class
1	5NXV	5nxv	3.50	high	2	8OM	5uwk	2.30	high
3	C1H	3ni5	33.47	high	4	WWV	3n3j	1.28	high
5	LMS	6c7w	3410.00	low	6	4KB	4z1k	5.22	high
7	OYQ	3oyq	10.30	high	8	MB2	4kuw	81.00	high
9	5EN	5drr	0.00	high	10	COX	2aw1	43.00	high
11	EL8	6g3o	0.58	high	12	R4C	4gr8	14.85	high

13	2Y6D	2y6d	79.00	high	14	NKX	3k2f	63.00	high
15	UDB	3u7l	52.40	high	16	GVR	2ves	20.00	high
17	FKE	6h36	0.45	high	18	2HOC	2hoc	0.65	high
19	J31	4ngp	10.99	high	20	1GD	4itp	63.64	high
21	3O1	4rfc	6.13	high	22	4D9W	4d9w	54.00	high
23	TI2	1qf0	42.00	high	24	WZA	2x7u	5340.00	low
25	2SD	3hkt	4100.00	low	26	3A2	3aho	88.90	high
27	99	3hyg	3324.00	low	28	MFS	3hkn	4594.21	low
29	IN9	1usn	18.00	high	30	OIR	1r1j	2.30	high
31	3OJ	3o0j	65.00	high	32	915	1yqy	24.00	high
33	9EB	5ny6	13.50	high	34	SMS	1eou	36.00	high
35	CFE	3lgg	0.10	high	36	0LX	4dv8	0.58	high
37	1ZFQ	1zfq	30.00	high	38	KLJ	4guy	10.00	high
39	S2O	4yyt	21000.00	low	40	J96	4ngn	28.10	high
41	9C2	5nw7	1700.00	low	42	S8A	5clu	78000.00	low
43	5DYF	5dyf	2300.00	low	44	720	2jew	57.41	high
45	OEV	3d50	17000.00	low	46	AHN	2afw	17000.00	low
47	I38	2qdt	6500.00	low	48	HQT	4q8y	2300000.00	low
49	O7U	3o7u	52.00	high	50	RTD	2m5d	90.00	high
51	LWY	3n2c	7500.00	low	52	5MH	4q7v	260000.00	low
53	6MH	4q7w	2300000.00	low	54	YHO	3dx1	265000.00	low

(c)

No.	Ligand name	PDB ID	Ki (nM)	Class	No.	Ligand name	PDB ID	Ki (nM)	Class
1	0K7	4dhl	33000	low	2	4SY	4zw6	1900	low
3	61	1a8t	1600	low	4	4T2	4zw8	1100	low
5	PC5	1p6e	1210000	low	6	JT6	2jt6	25	high
7	X16	4hwp	35.1909	high	8	M3S	3fcq	1700000	low
9	D66	3q43	43	high	10	AGF	8cpa	0.71	high
11	KIF	1ps3	5200000	low	12	EO7	6c7x	96.5588	high
13	TD3	3n9r	70	high	14	ZEB	1ctu	0.0012	high
15	CCB	1u3t	10000000	low	16	FC4	5j8z	5	high
17	24F	3elm	0.19	high	18	KLH	3rtt	7000	low
19	1B3	4hws	0.979796	high	20	ZAF	6cpa	0.003	high
21	FOT	2eg8	31800	low	22	PIU	1ze8	21	high
23	TM7	4mlx	650000	low	24	FXY	1u3w	40000	low
25	4WA	5byi	30.7	high	26	FMS	1bcd	2	high
27	51J	5aml	0.7	high	28	TH0	2osf	148000	low
29	HPR	2ada	0.0001	high	30	RS2	966c	23	high

31	56W	5d2r	1500	low	32	PN3	5jf8	1428	low
33	SXS	4cq0	8106	low	34	4RUZ	4ruz	23.1	high
35	IT5	3p5l	40.8	high	36	4FH	4q87	14000	low
37	LHY	1hee	4600	low	38	BIR	1r1h	1.2	high
39	5WM	5fdi	18.5	high	40	UHF	3u7n	11.2	high
41	45I	5am d	2.8	high	42	TQI	2y6c	10000	low
43	GOO	3dx4	76	high					

Table S5. Information on the 34 descriptors used in **Model 5.**

Descriptor names	description	Classes
ESP_balance parameter_Propgen_VAMP	Electrostatic potential energy	Electrical
BCUT_PEOE_3	Local distribution of charge values in PEOE	Adjacency and Distance Matrix Descriptors
petitjean	Value of (diameter - radius) / diameter.	
nBr	Number of bromine atoms	Atom Count Descriptor
nN	Number of nitrogen atoms	
AATS7s	Average Broto-Moreau autocorrelation - lag 7 / weighted by I-state	Autocorrelation descriptors
AATS8s	Average Broto-Moreau autocorrelation - lag 8 / weighted by I-state	
ATSC1s	Centered Broto-Moreau autocorrelation - lag 1 / weighted by I-state	
GATS5e	Geary autocorrelation - lag 5 / weighted by Sanderson electronegativities	
VE1_Dzs	Coefficient sum of the last eigenvector from Barysz matrix / weighted by I-state	Barysz Matrix Descriptor
BCUTp-1h	nlow highest polarizability weighted BCUTS	BCUT Descriptor
SpMax2_Bhm	Largest absolute eigenvalue of Burden modified matrix - n 2 / weighted by relative mass	Burden modified eigenvalue descriptor
ES_Count_ssNH	Calculate the E-state count for atom	Estate Keys

	type NH.	
IC1<br>MIC5	Information content index (neighborhood symmetry of 1-order)<br>Modified information content index (neighborhood symmetry of 5-order)	Information Content Descriptor
nAtomP	Number of atoms in the largest pi system	Largest Pi System Descriptor
HBA_Count	The number of hydrogen bond accepting groups in the molecule.	Molecular Property Counts
PEOE_VSA-0<br>PEOE_VSA+1<br>PEOE_VSA-4	Sum of vi where qi is in the range [-0.05,0.00).<br>Sum of vi where qi is in the range [0.05,0.10).<br>Sum of vi where qi is in the range [-0.25,-0.20).	Local charge descriptors
vsa_acc	Approximation to the sum of VDW surface areas (Å2) of pure hydrogen bond acceptors (not counting atoms that are both hydrogen bond donors and acceptors such as -OH).	Pharmacophore Feature Descriptors
SlogP_VSA3<br>SlogP_VSA1<br>SMR_VSA4<br>npr1<br>vsurf_EDmin3	Sum of vi such that Li is in (0,0.1].<br>Sum of vi such that Li is in (-0.4,-0.2].<br>Sum of vi such that Ri is in (0.39,0.44].<br>Normalized PMI ratio pmi1/pmi3.<br>Lowest hydrophobic energy	Subdivided Surface Areas
pmiX<br>pmiY	x component of the principal moment of inertia (external coordinates).<br>y component of the principal moment	Surface Area, Volume and Shape Descriptors

	of inertia (external coordinates).	
pmiZ	z component of the principal moment of inertia (external coordinates).	
JGI2	Mean topological charge index of order 2	Topological charge descriptors
JGI6	Mean topological charge index of order 6	
JGI8	Mean topological charge index of order 8	
JGI9	Mean topological charge index of order 9	
VR2_D	Normalized Randic-like eigenvector-based index from topological distance matrix	

Table S6. The interpretation and distribution of the eight important electronical-related descriptors in **Model 5**.

Descriptor names	μ(μ–1.96σ,μ+1.96σ) high activity	μ(μ–1.96σ,μ+1.96σ) low activity	Correlation	P value	Classes	Interpretation
JGI9	0.009 (-0.003,0.021)	0.004 (-0.006,0.014)	0.44	0	Adjacency and Distance Matrix Descriptors	Average Broto-Moreau autocorrelation - lag 8 / weighted by I-state
JGI8	0.011 (0.001,0.021)	0.006 (-0.01,0.022)	0.36	0		Local distribution of charge values in PEOE
HBA_Count	3.637 (0.178,7.096)	2.061 (-2.763,6.885)	0.35	0	Molecular Property Counts	Normalized Randic-like eigenvector-based index from topological distance matrix
PEOE_VSA-4	22.841 (-11.155,56.837)	11.374 (-13.565,36.313)	0.34	0	Local charge descriptors	Mean topological charge index of order 6
PEOE_VSA+1	57.195 (1.004,113.386)	38.923 (-11.261,89.107)	0.31	0		Geary autocorrelation - lag 5 / weighted by Sanderson electronegativities
PEOE_VSA-0	46.382 (-15.378,108.142)	29.62 (-18.892,78.132)	0.30	0		Mean topological charge index of order 9
nAtomP	11.311 (1.143,21.479)	7.316 (-0.206,14.838)	0.40	0	Largest Pi System	Average Broto-Moreau autocorrelation - lag 7 / weighted by I-state

					Descriptor	
BCUTp-1h	10.941 (8.45,13.432)	10.026 (7.355,12.697)	0.35	0	BCUT Descriptor	Centered Broto-Moreau autocorrelation - lag 1 / weighted by I-state

Table S7. The structure and activity of 10 compounds obtained by virtual screening for HDAC1.

Compd. No.	Structure	Inhibition Rate (%)	Compd. No.	Structure	Inhibition Rate# (%)
1	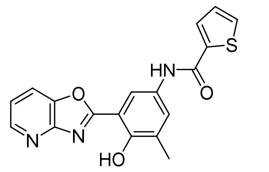	3.5 ± 2.3	6	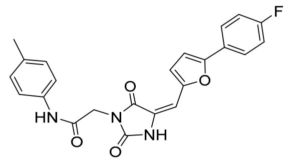	17.8 ± 3.0
2	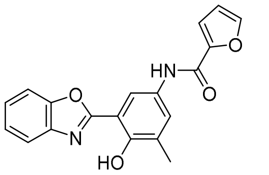	4.7 ± 0.2	7	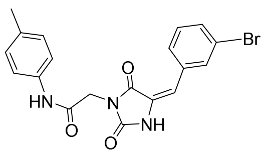	4.1 ± 1.8
3	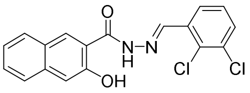	16.2 ± 5.5	8	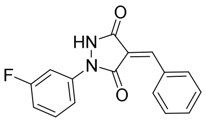	23.3 ± 1.8
4	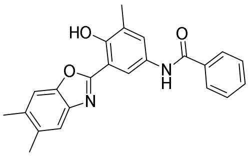	3.4 ± 3.8	9	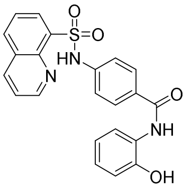	**99.4 ± 0.0**
5	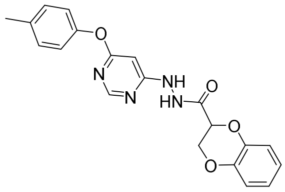	1.1 ± 7.4	10	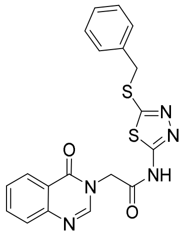	6.2 ± 0.2

The percentage inhibition was calculated from two measures under the concentration of 100 μM.
